# Increased anger and stress and heightened connectivity between IFG and vmPFC in victims during social interaction

**DOI:** 10.1038/s41598-024-57585-y

**Published:** 2024-04-11

**Authors:** Ann-Kristin Röhr, Nils Kohn, Rene Bergs, Benjamin Clemens, Angelika Lampert, Marc Spehr, Ute Habel, Lisa Wagels

**Affiliations:** 1 Department of Psychiatry, Psychotherapy and Psychosomatics, Uniklinik RWTH Aachen, Pauwelsstr. 30, 52074 Aachen, Germany; 2 Donders Institute, Centre for Cognitive Neuroimaging, Nijmegen, The Netherlands; 3 Jülich Aachen Research Alliance (JARA) - Translational Brain Medicine, Jülich, Germany; 4 Institute of Neurophysiology, Uniklinik RWTH, Aachen, Germany; 5 Department of Chemosensation, Institute for Biology II, RWTH Aachen University, 52074 Aachen, Germany; 6 Scientific Center for Neuropathic Pain Aachen – SCN Aachen, Uniklinik RWTH Aachen University, 52074 Aachen, Germany

**Keywords:** Victims of violence, Trans-diagnostic sample, Cyberball, Functional connectivity, BOLD brain activity, Human behaviour, Stress and resilience, Social behaviour

## Abstract

Self-identification as a victim of violence may lead to increased negative emotions and stress and thus, may change both structure and function of the underlying neural network(s). In a trans-diagnostic sample of individuals who identified themselves as victims of violence and a matched control group with no prior exposure to violence, we employed a social exclusion paradigm, the Cyberball task, to stimulate the re-experience of stress. Participants were partially excluded in the ball-tossing game without prior knowledge. We analyzed group differences in brain activity and functional connectivity during exclusion versus inclusion in exclusion-related regions. The victim group showed increased anger and stress levels during all conditions. Activation patterns during the task did not differ between groups but an enhanced functional connectivity between the IFG and the right vmPFC distinguished victims from controls during exclusion. This effect was driven by aberrant connectivity in victims during inclusion rather than exclusion, indicating that victimization affects emotional responses and inclusion-related brain connectivity rather than exclusion-related brain activity or connectivity. Victims may respond differently to the social context itself. Enhanced negative emotions and connectivity deviations during social inclusion may depict altered social processing and may thus affect social interactions.

## Introduction

Negative effects are well-documented for childhood maltreatment with consequences often persisting into adulthood^1,2^, influencing plasticity processes and functional brain networks^3^. In addition to physical or sexual violence, emotional abuse is associated with mental health problems in both childhood and adult life, predicting (social) anxiety, depression, and self-harm^4–6^.

**Figure 1:**
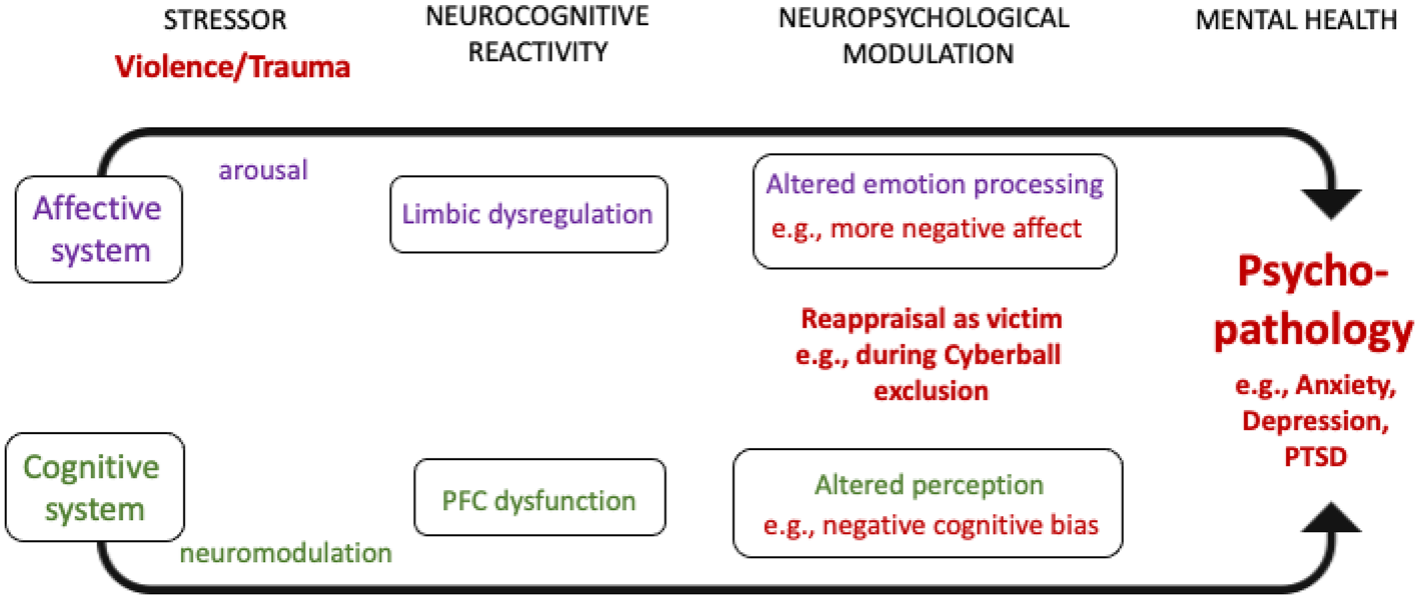
Adapted from Palamarchuk & Vaillancourt^7^. The experience of violence as a highly stressful life event can trigger a change in stress appraisal and reactivity through the convergence of the affective and cognitive systems, leading to dysfunctions and a cycle of psychological problems which reinforce the dysfunctional processing.

Past trauma such as violent experiences can influence how future stress is processed. As outlined in Fig. 1, stress, like experiencing violence can influence neural processing via affective and cognitive pathways, which modulate limbic and prefrontal cortex (PFC) dysfunction. As this may result in altered emotion processing and social perception, individuals may tend to reappraise new stressful situations as being re-victimized^7^. The processes are not specific to a mental disorder but describe a trans-diagnostic mechanism including changes in neural systems.

Evidence for a modified stress perception after violence can be found in various contexts. For example, war-related trauma in soldiers leads to a negative interpretation bias for emotional stimuli in these individuals^8^. The soldiers experienced a mild threat as overly negative and interpreted ambiguous situations negatively as well. Similarly, victims of interpersonal violence often perceive increased threat in social settings, even without direct harm being present^9^. Prior experiences of bullying determined the social stress reaction to exclusion in a sample of individuals with and without social anxiety disorder, even more so than their diagnosis^10^.

Altered stress processing after trauma is not only evident in future stressful situations but also in modified brain activity. When experiencing violence early in life, it is possible that the brain is rewired in salience detection and emotion valence circuits, which leads to different social threat processing in future adverse situations like social exclusion^11^. Furthermore, victimized adolescents exhibited altered brain activation in affect, reward and social pain processing regions during an acute ostracism situation. After the social exclusion experience, the individuals exhibited an increased risk-taking behaviour and functional activity suggested more effortful cognitive control^12^.

Hereby, the self-identification as a victim is important. Students who experienced bullying and labelled themselves as victims experienced poorer psychosocial functioning than those who did not adopt the label^13^. Similarly, the self-labelling as a “survivor” rather than as a victim has advantages. An online survey revealed that being labelled as a victim is more strongly associated with depression, post-traumatic stress disorder  (PTSD) and shameful emotions than a more rageful view on the offender from the perspective of a survivor^14^. In our study, we investigate individuals as victims who explicitly mention and retell a history of violence and label themselves as victims of violence.

Psychological consequences of violence are complex. Many survivors suffer from multiple mental health problems that benefit from a trans-diagnostic treatment approach^15–17^. In unison, stressful experiences like violence can be both a trigger and a catalyst for mental health problems^18^. We therefore chose a trans-diagnostic sample for realistic representation of the target group and included every form of victimization, emphasizing the subjective component of the individual feeling traumatized. This also improves the generalizability of our results as opposed to those in specific patient groups. With this approach, we aimed to investigate abuse-specific effects and isolate potential confounding factors like mental health disorders.

Violence-inflicted vulnerability is likely reinforced by alterations in the central nervous system compromising social functioning^19^. Changes in brain connectivity, independent of psychopathology, have been detected in veterans with early-life trauma compared to soldiers without adverse experiences during childhood^20^. The authors also noticed reduced functional connectivity between the amygdala, right parahippocampal and middle frontal gyrus in individuals who experienced interpersonal violence. Similar findings were reported for adolescent women who were previously exposed to or witnessed physical or sexual violence. They showed a decrease in dynamic resting state functional connectivity between amygdala and medial PFC compared to a control group^21^. Although research regarding brain activation and functional connectivity in adult self-identified victims is still sparse, there is some evidence that neural alterations in adult victims affect the stress system^22^.

Social exclusion is a strong stressor in healthy unburdened^23,24^ and even more in traumatized individuals^25^. In experimental settings, Cyberball mimics social exclusion and thus, induces social threat^26^. In this task, participants play a virtual ball tossing game with two confederates. During exclusion participants—uninformed—are mostly ignored by other players, which elicits feelings of anger and stress^27^. Increased anger reactions during Cyberball have been observed in adult patients with chronic depression^28^ and borderline personality disorder^29^. Furthermore, experiencing anger is associated with psychiatric symptoms in cyberbully-victims^30^. Thus, prior victimization may increase the negative effect of social exclusion on emotions, specifically anger.

In healthy participants, exclusion in the Cyberball task is consistently associated with bilateral activation of the ventral anterior cingulate cortex (ACC), extending towards the ventral and medial PFC^31^. A meta-analysis focusing on exclusion in Cyberball paradigms attests participation of the bilateral medial PFC and posterior cingulate cortices, right precuneus and ACC, left inferior frontal gyrus (IFG) and left orbitofrontal cortex (OFC)^32^. These typical neural activation patterns, however, exhibit individual variation depending on psychiatric psychopathology^33^. Depressed patients show heightened activity in the insula, amygdala and ventrolateral PFC during exclusion, among others correlating with low self-esteem^34^. It has not yet been investigated, if there are trans-diagnostic patterns associated to impaired emotion regulation or social stress processing in victims of violence.

Findings in children and juveniles point towards coherent patterns of increased neural sensitivity to social exclusion due to previous victimization^12^. In previously bullied adolescents, exclusion was associated with increased responses in the amygdala, parahippocampal gyrus, inferior frontal operculum and fusiform gyrus^35^ and reduced fronto-limbic connectivity^36^. Additionally, victimized girls showed an increased association between neural reactivity to exclusion and internalizing symptoms of depression and social anxiety^37^.

Regarding gender differences in mood changes after social exclusion, research shows mixed results. Some studies find no differences in response to social exclusion between females and males^23,38^, whereas others identify women to be more negatively affected^39^. Meta-analytic results^40^ do not suggest general moderating effects of gender in ostracism; however, differences might be small and specific to the outcome measure. While males seem to react with more retaliation after being excluded, females showed more displaced aggression^41^. We thus do not have a specific direction, but expect gender differences with regard to affective responses after being excluded.

To the best of our knowledge, there is no fMRI study on the processing of social exclusion in victimized adults. Since many victimized individuals suffer from mental health problems and complex psychological consequences, we chose a trans-diagnostic sample for realistic representation of the target group. Participants who self-identified as victims suffered from diverse forms of abuse including physical, sexual and emotional forms as well as economical violence. We aimed to investigate subjective emotion processing during Cyberball and functional activity and connectivity between task-relevant brain regions. As outlined in Fig. 1, we focused on a trans-diagnostic sample because complex affective and cognitive processes can foster diverse psychopathologies^7^. We hypothesized increased negative emotional reactions (anger, stress, teammate evaluation) and altered neuronal responses in the limbic system to social exclusion in the victim group. Specifically, dysfunctions in fronto-limbic brain connectivity could reflect deficits in emotion regulation via disrupted top-down processing of emotions and indicate heightened negative affect in victims during social stress^37^. We hypothesized disrupted connectivity between limbic areas and PFC regions similar to findings in adolescents.

## Methods

### Participants

For our analysis, we included 39 participants who self-identified as victims of violence (V, 19 men; mean age: 32.68 ± 9.69 years and 20 women; mean age: 34.4 ± 11.81 years) and 33 without a history of violence (NV, 15 men; mean age: 35.2 ± 11.28 years and 18 women; mean age: 33.33 ± 12.8 years) from a total of 84 (twelve excluded due to suboptimal quality of brain data). All participants had normal or corrected-to-normal vision, no contraindications against MR measurements, were fully right-handed [Edinburgh Handedness Inventory; Ref.^42^] and matched for a diagnosis of mental illness (see Table 1, “Results”) assessed by the Mini International Neuropsychiatric Interview [MINI; Ref.^43^]. V were recruited in a preceding study, NV were contacted through recruitment postings or were inpatients at the university hospital Aachen. Nobody had taken part in an experiment of social exclusion before. Prior to the MR measurement, participants were tested for verbal intelligence^44^, executive function^45^, verbal fluency and working memory^46^. V additionally completed subjective ratings onthe severity of their violent experiences, and a severity index was created based on the interview (see Supplemental Material).

**Table 1:** Prevalence of specific psychopathologies and types of violence exposure of male and female victims (V) and non-victims (NV).

Characteristics	V		NV		Significance at 95% confidence level		
	Women (20)	Men (19)	Women (18)	Men (15)			
					Group	Gender	Group × Gender
Psychopathology: MINI diagnosis	13	14 (n = 17)	11	10	0.401	0.288	Females: 0.804
							Males: 0.306
Psychotropic drugs	10	6	2	8	0.345	0.397	Females: 0.010
							Males: 0.201
BDI total scores	15	13 (n = 18)	7	11 (n = 14)	0.130	0.504	Females: 0.031
							Males: 0.949
Kind of abuse							
Physical	13	12	–	–	–	0.905	–
Sexual	8	4	–	–	–	0.200	–
Emotional	14	9	–	–	–	0.151	–
Economical	4	4	–	–	–	0.935	–
Multiple violence	20	2 (n = 18)	–	–	–	< 0.001	–
Childhood violence	12	14 (n = 18)	–	–	–	0.493	–
Psychopathology							
Affective disorders*	9	6	5	8	0.936	0.706	Females: 0.272
							Males: 0.201
Anxiety disorders*	8	7	0	0	< 0.001	0.961	Females: 0.003
							Males: 0.008
OCD*	1	0 (n = 17)	0	0	0.247	0.198	Females: 0.336
							Males: 0.170
PTSD	1	1 (n = 17)	1	0	0.345	0.274	Females: 0.939
							Males: 0.232
SUD* (Alcohol)	1	2 (n = 17)	0	0	0.091	0.214	Females: 0.336
							Males: 0.133
Psychotic disorder (past)	1 (n = 19)	1 (n = 17)	0	0	0.151	0.290	Females: 0.336
							Males: 0.232
Antisocial personality disorder	0 (n = 14)	1 (n = 15)	0	0	0.177	0.197	Females: NA
							Males: 0.211

Affective Disorders include diagnoses of acute Major Depressive Disorder, Melancholic Depression, Dysthymia, Hypomania and Mania; Anxiety Disorders include diagnoses of Panic Disorder, Agoraphobia, Social Phobia and General Anxiety Disorder; *OCD* Obsessive–Compulsive Disorder, *SUD* Substance Use Disorder.

### Ethical approval

Experimental procedures were performed in compliance with the latest version of the Code of Ethics of the World Medical Association [Declaration of Helsinki; Ref.^47^] and approved by the Ethics Committee of the Medical Faculty of the RWTH Aachen University. Participants gave written informed consent and received monetary compensation (80 Euros) as well as a full debriefing after participation.

### Procedure

Participants were blind about the real purpose of the task. They were instructed to engage in a virtual ball-tossing game intended to assess brain activation patterns during social interaction. Before the experiment, participants were introduced to two confederates (Mark and Nora) and then entered the test room. It was explained that the “teammates” would be placed in separate other rooms; however, in reality, they did not participate further in the study. Before and after the task, participants rated their emotional state using the positive and negative mood scale^48^.

The task was implemented using Presentation^®^ software (Version 16, www.neurobs.com) and presented by MR compatible video glasses (VisuaStimDigital; Resonance Technology, resolution: 800 × 600). Participants saw the virtual teammates located on the right and left side of the screen. A hand in the lower centre of the screen represented the participant. To throw the ball, one could press a button on an Ôher the left or the right teammate, respectively.

**Figure 2:**
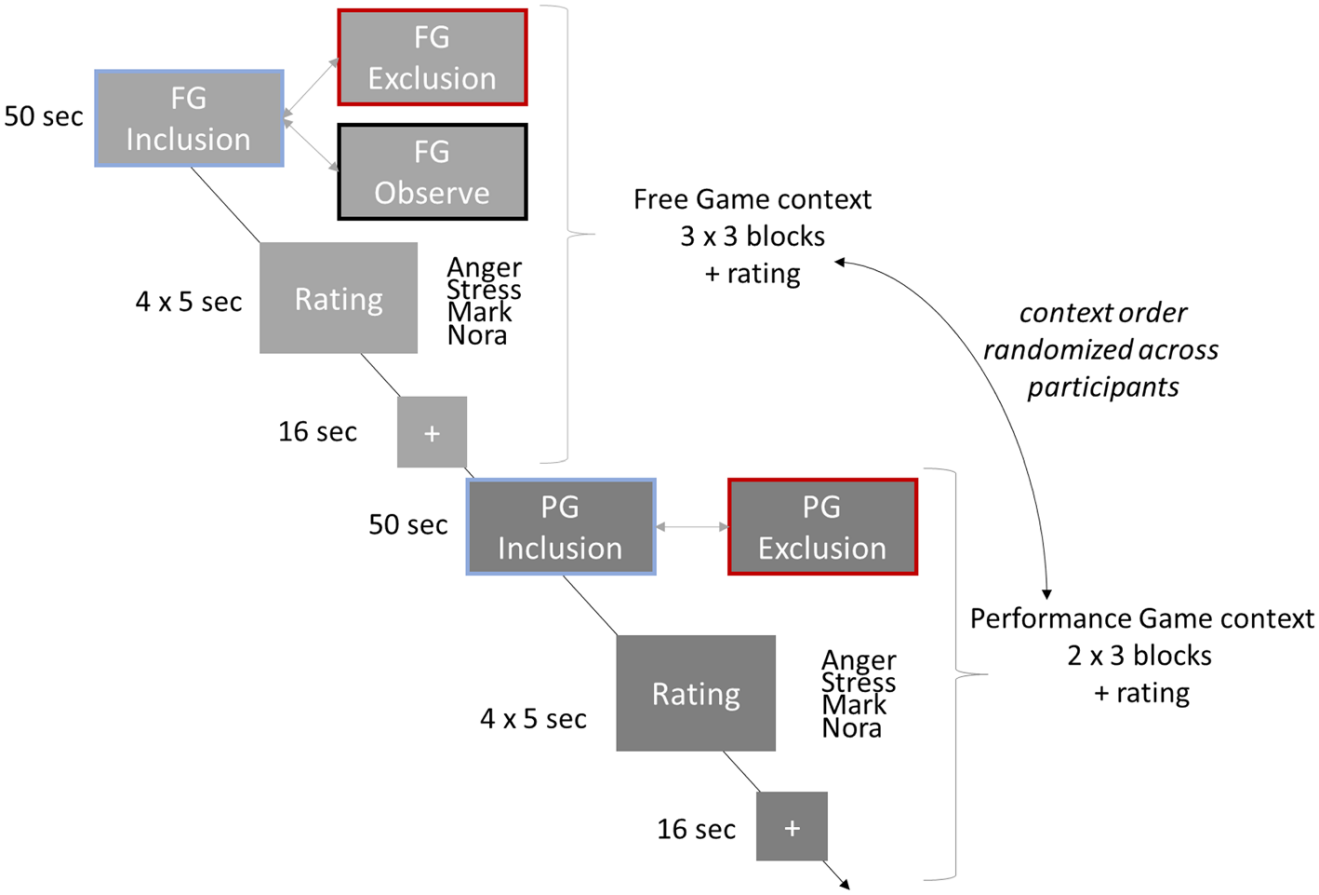
Schematic overview of the Cyberball gaming phases. The game started with an inclusion block either in the free game (FG) or in the performance game (PG) context. Afterwards, within each game context, inclusion and exclusion phases (and observe phases during FG) alternated randomly, followed by four ratings (anger, stress, fondness for Mark, fondness for Nora) on a nine-point Likert scale and a fixation cross before the next game block started. For simplification, we do not show the pre-phase of 12–16 s of inclusion during the exclusion blocks.

The fMRI task (for details, see Supplements), piloted by Wagels et al.^24^, comprised fifteen blocks (6 Performance Game (PG) blocks, 6 Free Game (FG) blocks, 3 Observation (O) blocks, Fig. 2). In O blocks, participants could not interact with the other players and were told that these blocks were used solely for technical reasons. O blocks were not part of our research questions.

Unknown to the participants, the game was divided into inclusion and exclusion phases. In exclusion, the supposed teammates stopped throwing the ball either after 12, 14, or 16 s (pseudo-randomized) resulting in exclusion phases averaging 36 seconds.

After every block, participants evaluated subjective perception of stress (“Do you feel stressed?”) and anger (“Do you feel angry?”) by shifting a bar with their finger on a 9-point Likert-like scale (1 = “not at all” and 9 = “extremely”). The third and fourth questions indirectly measured social bonding (“Do you like your teammates Mark/ Nora?”). Before the next block started, a fixation cross was presented for 16 seconds, serving as baseline.

After the task, participants filled out several questionnaires, including the Need-threat scale [NTS; Ref.^49^] and a credibility check. Further questionnaires were: Beck’s depression inventory [BDI-II; Ref.^50^], State Trait Anxiety Questionnaire [STAI; Ref.^51^], Brief Symptom Inventory^52^, Stress coping inventory^53^, Quality of life, Assessment of DSM-IV Personality Disorders Questionnaire^54^ and Emotion Regulation Questionnaire^55^. Furthermore, participants performed neuropsychological tests as described above.

### Statistical analysis of questionnaire data

Psychopathology ratings and ostracism questionnaire data of participants were compared per group (V, NV) and gender (men, women) by a univariate ANOVA using IBM SPSS Statistics for Macintosh, Version 23.0. Prevalence of violence exposure was compared between men and women in the V group by chi-squared test.

### Statistical analysis of behavioural data

Subjective ratings during the task, which were recorded separately for each game block, were summarized in mean scores for each individual according to context (FG, PG) and condition (IN, EX). Ratings for stress, anger and teammate evaluation were not normally distributed. We thus analysed data in a 2 × 2 within-subject design applying a generalized linear model [GLMZ; Ref.^56^] with gamma distribution and log link in R (Version 1.4.1717; 2021). We further added gender and group as between subject factors as well as task credibility, indicating whether participants believed the cover story or not.

### Acquisition and analyses of fMRI data

Imaging data were acquired using a Siemens 3 T Prisma scanner (Siemens AG; Erlangen, Germany) equipped with a 22-channel head matrix coil located in the Department of Psychiatry, Psychotherapy and Psychosomatics, University Hospital RWTH Aachen, Germany. Foam pads stabilized the head of the subject during the fMRI session. Each consisted of four functional runs (including two resting state measurements and a mood induction task after the final ratings, not reported here) and one anatomical run. One resting state measurement was performed before Cyberball. All other tasks and the anatomical run were performed afterwards. A time series of 553 functional images per participant was acquired, using a spin-echo EPI sequence with the following acquisition parameters: TR = 2000 ms; TE = 28 ms, flip angle = 77°, FOV = 192 * 192 mm, matrix size = 64 × 64 mm, 34 slices, voxel size = 3 × 3 × 3.75 mm^3^. Functional scans lasted about 18.5 min, including a pre-baseline (28 s). Structural scans were acquired using a T1-weighted MPRAGE sequence with the following acquisition parameters: TR = 2300, TE = 3.03 ms, flip angle = 9°, FOV = 256 * 256 mm, matrix size = 64 × 64 mm^2^; 176 slices, voxel size = 1 × 1 × 1 mm^3^.

Imaging data were analysed using SPM12 software (http://www.fil.ion.ucl.ac.uk/spm/). Participants who, from task beginning to task end, moved 5 mm or more (regarding displacement or rotation of their head) were excluded from the analysis (*n* = 9). Participants with displacement or rotation peaks over 3 mm were excluded as well. For data quality sufficiency, we also checked the global percentage signal change (PSC), which has been suggested to be a good information criterion of imaging quality^57^. The images of the time-series were realigned with a two-pass procedure, with the first image (first pass) and the mean image (second pass) as references. Co-registration of each anatomical scan to its mean EPI was performed, which was subsequently used to determine spatial normalization parameters by means of the unified segmentation approach^58^. These normalization parameters were applied to the functional scans, thus transforming the time-series into the standard space defined by the Montreal Neurological Institute (MNI). During normalization, all images were resampled to a voxel size of 2 × 2 × 2 mm^3^. Afterwards, images were smoothed using an isotropic Gaussian kernel of 8 mm full-width-at-half-maximum. For individual time-series, 6 parameters modelling the realignment parameters (x, y, z translation, rotation) and the PSC were included as regressors. Individual time-series were analysed (first-level) within the framework of the general linear model (GLM). Seven boxcar functions (PG EX, PG IN, FG EX, FG IN, O, one for the 12–16 s preliminary phase of exclusion in EX blocks and one modelling the ratings) were convolved with the canonical hemodynamic response function (HRF) implemented in SPM12. Furthermore, we used the default high-pass filter with a cut-off of 128 s (0.008 Hz). On the second level, a fixed effects analysis was conducted applying a full factorial model with a 2 × 2 × 2 design. Context (PG, FG) and condition (EX, IN) were within-subject factors and group (V, NV) between-subject factor.

### Functional connectivity analyses

ROI-to-ROI functional connectivity was calculated using CONN functional connectivity toolbox v18a [http://www.nitrc.org/projects/conn; Ref.^59^]. Functional images were realigned and unwarped to estimate and correct subject motion. Functional and structural centering enabled the translation according to both axes. After slice timing and outlier detection (ART-based identification of outliers), data were segmented, normalized, and smoothed (spatial convolution with 8 mm Gaussian kernel). Denoising was performed by regressing noise components from white matter and cerebrospinal fluid to account for potential confounding effects. Furthermore, six motion parameters and their derivatives as well as squares of the six motion parameters, their derivatives and scrubbing parameters were modeled as regressors of no interest. Task regressors were modeled parallel to the BOLD activation GLM (see previous section). We did not apply global signal regression and de-spiking, but linear detrending, before regression. The time series were band-pass filtered to retain signals between 0.008 and 0.09 Hz to reduce susceptibility to physiological noise^60,61^.

For the ROI-to-ROI connectivity analysis, we developed masks based on the meta-analysis by Vijayakumar and colleagues^32^, which identified Cyberball-specific brain coordinates. We refrained from defining masks by our own exclusion > inclusion contrast in order to avoid potential circularity. For each mask, a sphere of five mm around the respective peak was applied. If two peaks were reported for a region only the first peak was modelled as a ROI (for an overview of ROIs see Supplemental Table 7). At the single-subject level, we calculated generalized psychophysiological interactions (gPPI). In short, the averaged BOLD time-course of each respective ROI was extracted depicting the physiological regressor. PPI regressors for each condition were created by calculating the element-by-element product between the physiological regressor and the psychological regressor (FG EX, FG IN, PG EX, PG IN, O). The correlation of each time-course of one ROI was correlated with the PPI regressor of another and the resulting betas were converted to z-scores using the Fisher’s z-transformation. On group level, we calculated directed contrasts of the connectivity between all ROIs and included as between-subjects factor group, gender, anxiety disorders, and the evaluated severity of violence. Connectivity analyses for the main effects of condition (EX vs. IN), context (FG vs. PG), group (V vs. NV) and gender (men vs. women) as well as the interaction of group (V vs. NV) and condition (EX vs. IN) and anxiety disorders on the EX > IN contrast were conducted.

## Results

### Psychopathology demographics

Table 1 depicts a group comparison of mental health characteristics and for V, the gender comparison regarding violence categories. For psychotropic drugs, 50% of women in the V group, compared to only 11% of women in the NV group, took any kind of medication. Furthermore, 40% of women and 36.8% of men in the V group, compared to no participant in the NV group, suffered from an anxiety disorder (as a comorbid diagnosis).

Women in the V group were significantly more often exposed to multiple violent events compared to men.

### Ostracism questionnaire data

The NTS sum score differed significantly between the V and the NV groups (Table 2). In the subscales of the NTS, the belonging scale as well as the meaningful existence scale were significantly different between victims and non-victims. For the STAI, we found a significant group difference at the trait level between V and NV.

**Table 2:** Ostracism measures (mean and standard error of the mean) between male and female V and NV.

Ostracism questionnaires	V		NV		Significance at 95% confidence level		
	Women	Men	Women	Men			
					Group	Gender	Group × Gender
Rejection sensitivity	10.81 ± 1.00 (n = 16)	12.63 ± 1.34 (n = 9)	10.47 ± 1.11 (n = 13)	11.02 ± 1.16 (n = 12)	0.404	0.313	0.587
NTS* Overall	9.83 ± 0.53 (n = 19)	10.38 ± 0.56 (n = 17)	11.53 ± 0.55	11.67 ± 0.60	0.010	0.543	0.714
NTS*: Belonging	2.47 ± 0.21 (n = 19)	2.54 ± 0.22 (n = 17)	3.09 ± 0.22	3.08 ± 0.24	0.012	0.896	0.864
NTS*: Self-esteem	2.8 ± 0.18 (n = 19)	3.09 ± 0.20 (n = 17)	3.04 ± 0.19	3.13 ± 0.21	0.467	0.335	0.619
NTS*: Meaningful existence	2.64 ± 0.020 (n = 19)	2.98 ± 0.21 (n = 17)	3.46 ± 0.20	3.47 ± 0.22	0.002	0.405	0.436
NTS*: Control	1.82 ± 0.13 (n = 19)	1.78 ± 0.14 (n = 17)	1.94 ± 0.13	1.99 ± 0.15	0.226	0.993	0.751
STAI*: state anxiety	42.35 ± 2.78	34.58 ± 2.85	33.17 ± 2.93	33.47 ± 3.20	0.085	0.209	0.175
STAI*: trait anxiety	47.35 ± 3.53	41.68 ± 3.62	35.11 ± 3.72	37.27 ± 4.07	0.029	0.640	0.299

*NTS* need-threat scale, *STAI* state-trait anxiety inventory.

### Behavioural data

**Figure 3:**
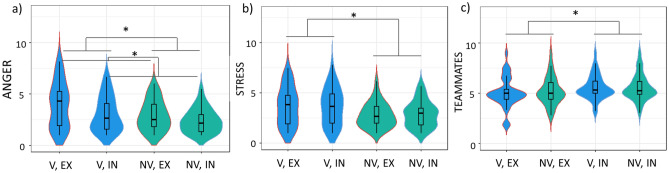
Violin plots for (**a**) anger (**b**) stress and (**c**) teammate ratings (on a scale from 1 to 9) illustrating the kernel probability density, i.e. the width of the shaded area represents the proportion of the data located at that position, including a box plot with depiction of the median in victims compared to non-victims separately for exclusion vs. inclusion. *Significance is indicated at p < 0.05.

The analysis of anger ratings revealed significant effects of condition, context, group and gender (Table 3). Anger ratings were higher during exclusion compared to inclusion (*F* (1, 278) = 11.65, *p* < 0.001, see Fig. 3a) and in PG compared to FG (*F* (1, 277) = 6.6, *p* = 0.01). Moreover, V were angrier than NV (*F* (1, 276) = 17.53, *p* < 0.001, Fig. 3a) and women scored higher on subjective anger than men (*F* (1, 274) = 8.81, *p* = 0.003). In the GLMZ on subjective stress ratings, main effects of context, group and gender were observed (Table 3). PG was rated as more stressful than FG (*F* (1, 277) = 12.97, *p* < 0.001) and V indicated more stress than NV (*F* (1, 276) = 11.36, *p* < 0.001, Fig. 3b). Additionally, women indicated more stress than men (*F *(1, 274) = 6.14, *p* = 0.014). Teammate evaluation between participants and the ostensible team players differed depending on condition, gender, and the participants’ belief in the cover story *(F* (1, 275) = 12.17, *p* < 0.001, Fig. 3c, Table 3). During the IN condition, positive teammate evaluation was higher compared to EX (*F* (1, 278) = 8.09, *p* = 0.005) and women indicated to like their teammates more than men (*F* (1, 274) = 8.67, *p* = 0.004).

**Table 3:** Significant main effects of the Generalized Linear Model for participants’ ratings of anger, stress and teammates.

	Df	Res. deviance	DF	Res. deviance	*F*	*p*
Anger						
Condition	1	3.92	278	112.36	11.65	< 0.001
Context	1	2.22	277	110.14	6.6	0.01
Group	1	5.9	276	104.24	17.53	< 0.001
Gender	1	2.96	274	101.17	8.81	0.003
Stress						
Context	1	4.25	277	106.71	12.97	< 0.001
Group	1	3.72	276	102.99	11.36	< 0.001
Gender	1	2.01	274	100.98	6.14	0.014
Teammates						
Condition	1	0.54	278	22.23	8.09	0.005
Gender	1	0.58	274	20.81	8.67	0.004
Task credibility	1	0.81	275	21.39	12.17	< 0.001

For a full overview of all effects please see Supplements.

### fMRI data

**Figure 4:**
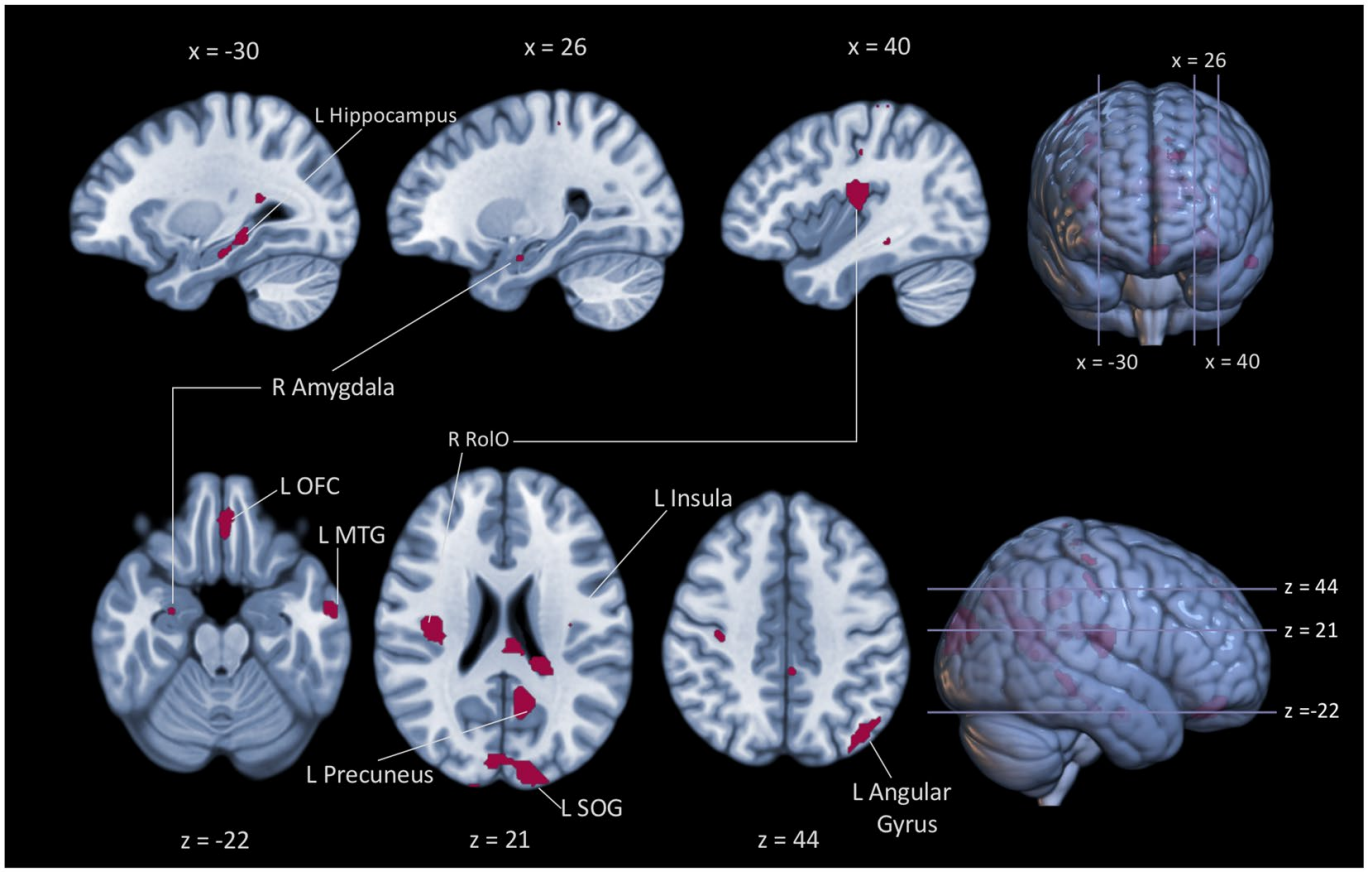
Significant activation clusters of the BOLD activation *t*-contrast EX > IN at voxel level FWE corrected threshold of *p* < 0.05 is superimposed on an MNI template brain (61) in fuchsia. On the right hand, cortical surface is displayed with the respective cutout slices that are displayed to the left. *L* left hemisphere, *R* right hemisphere. *OFC* Orbitofrontal Cortex, *MTG* Middle Temporal Gyrus, *RolO* Rolandic Operculum, *SOG* Superior Occipital Gyrus (hOc3d).

All results for the brain activity analysis were corrected for multiple comparisons on a whole brain FWE voxel level corrected threshold of *p* < 0.05 and a minimum cluster size of five voxels. Significant effects of EX > IN are depicted in Fig. 4 and Table 4 (for IN > EX, see Supplements). Context effects were significant as well (FG vs. PG; see Supplements). Neither the main effect of group (V vs. NV) nor any interaction revealed a significant effect.

**Table 4:** Anatomical labeling and MNI coordinates for the peak voxel of significant clusters of the EX > IN BOLD activation t-contrast during the Cyberball Game.

Anatomical region	x	y	z	*t*	k
L hOc3d (Superior Occ. Gyrus, SOG)	− 16	− 100	22	7.83	462
L precuneus	− 10	− 56	22	6.81	380
L angular gyrus	− 44	− 74	44	7.88	304
R rolandic operculum (RolO)	40	− 16	18	10.05	250
L midcingulate cortex	− 4	− 40	40	6.56	156
L hippocampus	− 34	− 34	− 10	6.50	143
L rectal gyrus (L OFC)	− 4	44	− 20	6.06	138
L middle temporal gyrus (L MTG)	− 58	− 6	− 22	5.55	56
R precentral gyrus	36	− 20	44	5.39	44
R hippocampus	34	− 38	− 4	5.77	44
L superior frontal gyrus	− 16	52	40	5.60	25
R postcentral gyrus	48	− 24	60	5.88	15
L insula lobe (L Insula)	− 36	− 14	20	5.36	14
R calcarine gyrus	30	− 52	8	4.96	9
L superior frontal gyrus	− 10	46	48	5.76	8
R hOc2	18	− 100	22	5.12	8
R basolateral amygdala (R Amygdala)	26	− 8	− 22	5.02	8
R postcentral gyrus	36	− 30	70	5.23	7

x,y,z = MNI coordinates for the peak voxel, t = t value, k = number of voxels in each cluster.

*L* left hemisphere, *R* right hemisphere. Only gray matter is included in table.

### Functional connectivity

For the functional connectivity analyses we used ROIs based on the meta-review by Vijayakumar et al.^32^, deploying 29 studies and N = 857, limiting the number to one ROI per brain area (selecting the larger cluster). On a group level, the functional connectivity analysis of the correlation between the condition- specific time series of a ROI and independent time series of another ROI showed significantly different connectivity for the EX > IN contrast. Connectivity decreased towards EX (see Table 5), except for the left ventromedial PFC cortex and the left lateral OFC, between which connectivity increased during EX. This connectivity change was significant only at seed-level but not analysis-level correction. There were no significantly different connectivity patterns for the main effects FG vs. PG, V vs. NV and men vs. women, even at uncorrected threshold.

**Table 5:** Statistics of significant ROI-to-ROI connectivity changes comparing EX > IN.

Contrast	ROI-to-ROI connection	*t*	Uncorrected ROI-to-ROI connection	Seed-level, ROI-to-ROI connection FDR corr. *p*	Analysis-level, ROI-to-ROI connection FDR corr. *p*
Condition contrast (EX > IN)	L IFG—R Precentral Gyrus	− 3.87	0.0002	0.0017	0.0051
	L IFG—R Precuneus	− 3.28	0.0016	0.0056	0.0134
	L IFG—R vmPFC	− 3.52	0.0008	0.0053	0.0106
	R Precuneus—lateral L OFC	− 3.91	0.0002	0.0014	0.0051
	L vmPFC—lateral L OFC	2.58	0.0121	0.0422	n.s.*
V > NV during EX > IN	L IFG—R vmPFC	3.15	0.0024	0.017	n.s.*
V during EX > IN	R Precuneus—lateral L OFC	− 2.95	0.0054	0.0379	n.s.*
NV during EX > IN	L IFG—R Precentral Gyrus	− 2.90	0.0067	0.024	n.s.*
	L IFG—R Precuneus	− 2.87	0.0073	0.024	n.s.*
	L IFG—R vmPFC	− 2.72	0.0104	0.024	n.s.*
	L IFG—R sgACC	− 2.71	0.0108	0.029	n.s.*
V > NV during IN	R Precuneus—R sgACC	− 2.66	0.0097	0.0435	n.s.*
	R Precuneus—L vmPFC	− 2.72	0.0082	0.0435	n.s.*
	L vmPFC—R sgACC	− 2.64	0.0103	0.0465	n.s.*
Female V > NV during EX > IN	L IFG—R vmPFC	3.35	0.0019	0.0193	n.s.*
Participants with AXD vs. participants with no AXD	L IFG—L vmPFC	3.38	0.0012	0.0084	n.s.*
	L IFG—R vmPFC	3.03	0.0034	0.0103	n.s.*
	L IFG—ventral ACC	2.94	0.0044	0.0103	n.s.*
	Lateral L OFC—L vmPFC	2.60	0.0114	0.0455	n.s.*
	Lateral L OFC—R vmPFC	2.55	0.0130	0.0455	n.s.*

*Connections significant at the seed-level FDR correction failed to reach significance at analysis-level correction. *L* left, *R* right. Only grey matter is included in table. *SgACC* subgenual Anterior Cingulate Cortex, *vmPFC* Ventromedial Prefrontal Cortex, *OFC* Orbitofrontal Cortex, *IFG* Inferior Frontal Gyrus, *AXD* Anxiety Disorder.

**Figure 5:**
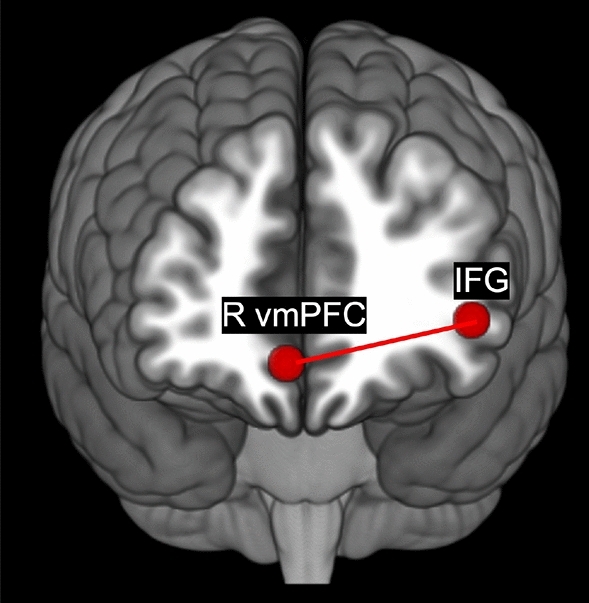
Victims compared to non-victims engage the right vmPFC-IFG connection more in exclusion than inclusion.

Interaction contrasts were only significant at seed-level correction. The interaction contrast of V vs. NV group by condition was significant for the connection between the right vmPFC and the left IFG. In V relative to NV, the right vmPFC- left IFG connectivity in difference of EX > IN was higher (Fig. 5).

**Figure 6:**
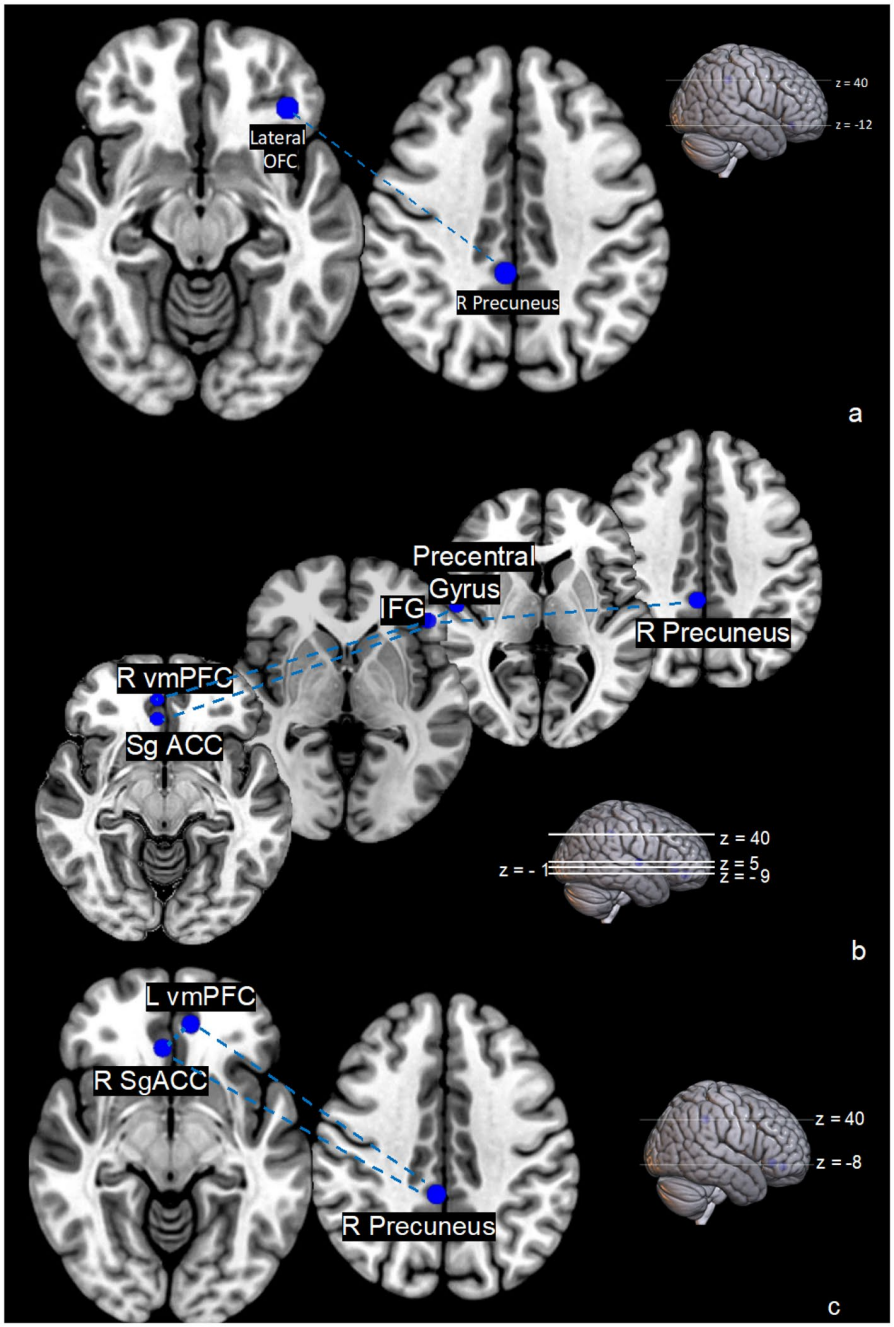
Connectivity in (**a**) victims during exclusion compared to inclusion, (**b**) non-victims during exclusion compared to inclusion (**c**) victims compared to non-victims during inclusion. Blue connections depict connectivity decreases.

Post hoc tests comparing EX to IN in the V group showed a significantly decreased connectivity in exclusion between right precuneus and left lateral OFC (Fig. 6a) but no significant in- or decrease of connectivity in the right vmPFC and left IFG pair. In the NV group, only node pairs in which connectivity decreased (Table 5) were identified including the right vmPFC—left IFG connection. In addition, in the NV group the connectivity between left IFG and the right precuneus, precentral gyrus and right sgACC was decreased in EX compared to IN (Fig. 6b). No significant connectivity differences between the V and NV group were found during EX. However, in the V group during IN, three nodes (right precuneus, right sgACC and left vmPFC) showed decreased connectivity among each other compared to the NV group (Table 5, Fig. 6c).

Sub-analyses on EX over IN showed connectivity differences at seed level correction only in female participants. Similar to the whole-group analysis, the connection between the left IFG and right vmPFC was increased. No significant connectivity difference was found in male V versus NV. Connectivity between the left IFG and right vmPFC was also increased in individuals with anxiety disorder compared to those without. Further connectivity differences in these groups included those between the left IFG and left vmPFC, as well as the ventral ACC and the left lateral OFC with the left and right vmPFC, respectively.

The severity index did not yield any results regarding changed connectivity in our sample.

## Discussion

Victims of violence (V group) experience negative effects on their emotional responses during socially stressful situations. As suggested by other studies^36,37^ and related to deviations in the neural system, social exclusion might be more harmful for previously victimized individuals. This is in line with Palamarchuk and Vaillancourt^7^, and we here applied a trans-diagnostic model to explain enhanced stress reactions after being victimized due to changes in cognitive and emotional systems. Our study highlights altered functional connectivity between regions related to the affective system and frontal areas in V, predominantly associated with differences during the inclusion period. Our results do not suggest homogeneous changes in brain activity as a consequence of victimization, which might be due to changes in different systems such as emotional reactivity or cognitive appraisal. The results do not indicate that the severity of violence influences brain activity or emotional responsiveness, thereby substantiating that it is the subjectively perceived victim status rather than the specific experience that modifies perception and neural connectivity. Finally, although the results document a trans-diagnostic phenomenon largely independent of a specific mental health disorder, pathological symptoms were stronger and anxiety disorders were more prevalent in the V group. This may contribute to the findings on a neural level and to the emotional ratings during the experience of a social exclusion scenario.

### Group differences of our sample: victimization and mental health

The V group was heterogeneous regarding both the type of violence and their current (mental) health status. Compared to NV, anxiety was higher in V, which is in line with others finding greater levels of social anxiety in peer-victimized youth^62,63^. Further measures of mental health status largely matched between V and NV, thereby minimizing the influence of specific pathologies when comparing the groups. Importantly, all participants in the V group subjectively felt victimized whereas none of the participants in the NV group did. Hereby, we compared two heterogeneous groups that differed specifically in their self-perception of victimization despite a broad definition of violence including physical, emotional, sexual and financially related violence. We thereby aimed to emphasize the subjective component of the individual and their identification as a victim instead of weighting the severity of the event(s). Previous research indicated that the specific traumatic event might not lead to a differentiated effect^64^, which justified the inclusion of all types of experienced violence.

### Differences in emotions and functional connectivity during inclusion

Our data revealed emotional and functional connectivity specificities in V who showed a stronger experience of anger and stress during exclusion and surprisingly also during inclusion periods in the Cyberball task. Despite the aforementioned reaction and previous evidence of differential brain activation towards exclusion in patient groups^34,65^ and young victimized individuals^35^, our results did not support brain activation differences between adult V and NV in response to social exclusion. Activity patterns might be similar in adults during the Cyberball game or the large heterogeneity within each group may obscure differences in potential subgroups.

In contrast, the functional brain connectivity between the vmPFC and the left IFG in V and NV differed in the inclusion but not exclusion. In NV but not V, connectivity decreased in the exclusion compared to inclusion. Moreover, connectivity between groups only differed during inclusion. This aberrant pattern may accompany a less effective emotion regulation^66^, here indicating problems of V to regulate emotions during functional social interactions or to have difficulties in functional situations after exclusion. V might have exerted more effort already during inclusion to control negative emotions.

In summary, our results do not indicate that exclusion is more detrimental to victims of violence. Instead, victims in general are more burdened than NV and show more anger and stress throughout the whole paradigm, impacting the exclusion as well. Victims may not be especially vulnerable for social exclusion, but this situation might be one of many that burdens victims and shows their higher vulnerabilities for stress situations in general. We see two alternative explanations for this: victims in general could be more burdened from the beginning and therefore perceive social interactions as debilitating. Secondly, the exclusion could have been more detrimental to victims, but they ruminated about the situation even during inclusion and had difficulties returning to a neutral baseline. Additionally, we observed functional brain differences, not regarding activity but functional connectivity, which emphasizes that there are indeed subtle differences in our trans-diagnostic victim group compared to the NV group.

### Social inclusion reveals neural vulnerability in the DMN

Our results indicate that it is not the exclusion that is more stressful for individuals who have experienced violent trauma, but the inclusion period. On the emotional level, we find V experiencing heightened anger and stress during inclusion, and not only during the typically stressful period of exclusion. During inclusion, our V group might have ruminated unable to return to a more neutral neural baseline, unlike NV. Alternatively, the V group might have had different social expectations and even felt rejected in the inclusion condition, comparable to some individuals with borderline personality disorder who only feel less negative emotions in an over-inclusion-situation^67^. In our group, this might reflect a distorted self-view of being the victim and perceiving others as violent and rejecting.

The ROIs implicated in V during inclusion (vmPFC, precuneus and sgACC) are part of the so-called Default-Mode Network (DMN), which is involved in thinking about oneself and others^68,69^. McIver et al.^36^ who also observed differences in fronto-limbic functional connectivity throughout exclusion and inclusion in peer-victimized adolescents, speculate that victimized adolescents may anticipate negative feelings already during inclusion because of their pessimistic self-reference. Other findings on the vmPFC suggest that this region may be particularly important during explicit expressions of subjective valuations^70^. After the experience of exclusion, in the inclusion period, participants may consider the relevance of the exclusion for themselves for example by processing who is including them now. In V accordingly, the perception of stress from the exclusion might be processed in inclusion. Similarly, the exclusion situation might be less meaningful, because the entire situation is perceived as stressful and still contemplated about during inclusion.

We cannot directly deduce meaning to the single regions implicated in functional connectivity differences here, however, the vmPFC and its connections seem to contribute to processing emotions during social interaction^71^ while connectivity between the sgACC and other nodes of the DMN seems aberrant in depression^72^. The precuneus plays a key role in self-perspective taking^73^, which is important for social processes.

Emotion generation may differ between V and NV due to a social bias, as seen in some patient groups during social inclusion^67,74^. Individuals with social anxiety more strongly anticipate exclusion and patients with borderline personality perceive exclusion even when being included. In victims, the interpretation of cues seems to be biased towards a focus on more negative stimuli or a maladaptive interpretation of attitudes and situations^75^. Domsalla et al.^76^ coined the term of hypermentalizing that is attributing meaning to social interactions which is not substantiated and intended. Thus, hypervigilance in trauma victims^77^ potentially biases social cognition during interactions. Similar to our findings adults victimized by peers showed a general avoidance towards social interactions and not only towards threats^78^. Other studies showed similar biases in victims of interpersonal violence and war-traumatized individuals^8,9^. Importantly, it remains unclear whether the vulnerability of a social bias is a direct consequence to experienced violence or whether the vulnerability makes people more prone to becoming a victim due to unintended different behavior^75^. Alternatively, individuals identify themselves as victims instead of survivors more frequently because they have a negative interpretation bias.

### Differences between task conditions

Comparing exclusion and inclusion, several Cyberball specific regions that are associated with emotion regulation and social evaluation processes (e.g., IFG, vmPFC, precentral gyrus and precuneus; Ref.^32^) showed both altered activity and altered connectivity, as we expected.

During exclusion, we observed increased activation in the OFC, the operculum, insula and left MTG, key regions implicated in social exclusion during the modified Cyberball paradigm^24,32,65^. Regardless of diagnosis or victim status, key regions associated with various large-scale brain networks were activated when participants faced social exclusion, supporting the robustness of the participating regions in processing social exclusion. The missing differences in our sample between V and NV regarding brain activation strength or patterns, as further indicated by emotional changes, are not a result of an ineffective simulation of social exclusion. Given the finding of increased negative affect during exclusion and a more negative evaluation of the team members, we conclude that our task successfully influenced our participants to experience mild social stress during our experimental exclusion.

### Anxiety

Anxiety, in addition to depression, was one of the most salient psychopathological symptoms in our sample and differed between the V and NV group. Individuals with an anxiety disorder showed a positive connectivity between the IFG and bilateral vmPFC, as well as the ventral ACC, compared to individuals without an anxiety disorder. Cha et al.^79^ proposed that the IFG serves as a threat informant to the vmPFC, which in turn inhibits the amygdala. In their study, clinically anxious individuals exhibited altered connectivity between the IFG and the vmPFC when anticipating an electric shock. In our sample, the presence of an anxiety disorder could modulate our results and partly explain the divergent connectivity found in the victim group. Nonetheless, trauma may have led to enhanced anxiety and neuronal changes. Anxieties connected to victimization should therefore be a focus of research on neural changes. Interventions, such as neurofeedback targeting the IFG and vmPFC, may offer the potential to improve anxiety-related problems in victims of violence.

### Gender

In our study, women were more reactive regarding stress and anger after the Cyberball manipulation. They experienced more anger and stress but also better social bonding with their purported teammates. This is in line with other findings^41^. In our sample, one reason for women being especially sensitive to social exclusion might be their compromised mental health. A study by Weik and colleagues found a dampened cortisol response to a stress task in women but not in men after social exclusion^80^. Further research has yet to determine whether women might be more susceptible to the consequences of ostracism when their health is compromised.

### Strengths and limitations

With this trans-diagnostic sample, we assembled a representative population sample with a large heterogeneity in terms of pathology, controlling for effects of (mental) disorder status. Thus, our results are strengthened and interpretations regarding the consequences of violence exposure are largely independent of psychopathology. Our research stands out for its use of a trans-diagnostic sample and its emphasis on the subjective experiences of individuals who feel victimized by a range of abusive and violent situations. However, high heterogeneity is induced, potentially weakening results during group comparisons and making it not fully possible to control for all disorders and symptoms. In this study, participants included in the victim group experienced diverse traumatization (childhood, multiple times, mild to severe), which, given the current sample size, precludes analysis of exposure-specific patterns which are certainly of importance. Future research should address the possible implications of different aspects of experienced violence and how these affect neural and emotional processing in larger sample sizes to counteract lack of power.

On the emotional level, we found that anger and stress were increased in victims of violence. Although we expected this, one must be cautious concerning the reliability of this finding. We only measured anger and stress with one item, respectively, and did not apply a more extensive item battery around the construct of these emotions.

We wanted to be complete and sensitive in the description of our fMRI results and therefore applied family-wise error correction, which is conservative and limits the reliability of small clusters.

Our results do not indicate a specific detrimental effect from being excluded in individuals perceiving themselves as victims of violence. Instead, V in general seem to be more burdened than NV and show more anger and stress throughout the whole paradigm, having an impact on the exclusion as well. Therefore, future studies with a group design and victims in inclusion vs. exclusion are needed.

### Conclusion

Our results provide a first insight into the subtle alterations in brain circuitry, specifically into the functional connectivity during social exclusion and inclusion for individuals previously exposed to violence. Overall, our data do not support a general effect of identifying as a victim of violence on the BOLD functional activation as a function of exclusion in the Cyberball game. However, we identified functional connectivity changes stemming from differences between V and NV in the inclusion. Altered connectivity in V may contribute to increased stress and anger observed in V during social interactions. We see multiple explanations for this and future studies have to find out, whether victims are in general more burdened and depleted by social situations or if they need more time to return to a neutral baseline after stress. Our findings may also highlight the need for general social interaction trainings for individuals who have been exposed to violence and for social resources during trauma therapy.

### Supplementary Information

Supplementary Information.

## Data Availability

Due to data protection of the individual, raw data remains confidential. Our code for the analysis of questionnaire and behavioural data, as well as for functional activation is available upon request (please send an E-Mail to the corresponding author AR if you are interested in our analysis code).

## References

[1] Sourander A (2016). Association of bullying behavior at 8 years of age and use of specialized services for psychiatric disorders by 29 years of age. JAMA Psychiat..

[2] Kisely S (2018). Child maltreatment and mental health problems in adulthood: birth cohort study. Br. J. Psychiatry.

[3] Holz NE (2023). Early social adversity, altered brain functional connectivity, and Mental Health. Biol. Psychiatry.

[4] Lereya ST, Copeland WE, Costello EJ, Wolke D (2015). Adult mental health consequences of peer bullying and maltreatment in childhood: Two cohorts in two countries. Lancet Psychiatry.

[5] Spencer C (2019). Mental health factors and intimate partner violence perpetration and victimization: A meta-analysis. Psychol. Violence.

[6] Turner HA, Finkelhor D, Ormrod R (2006). The effect of lifetime victimization on the mental health of children and adolescents. Soc. Sci. Med..

[7] Palamarchuk IS, Vaillancourt T (2022). Integrative brain dynamics in childhood bullying victimization: Cognitive and emotional convergence associated with stress psychopathology. Front. Integr. Neurosci..

[8] Gebhardt C, Alliger-Horn C, Mitte K, Glaesmer H (2017). All-or-nothing thinking: The processing of emotional expressions in traumatized post-deployment soldiers. J. Anxiety Disord..

[9] Elwood LS, Williams NL, Olatunji BO, Lohr JM (2007). Interpretation biases in victims and non-victims of interpersonal trauma and their relation to symptom development. J. Anxiety Disord..

[10] Iffland B, Sansen LM, Catani C, Neuner F (2014). The trauma of peer abuse: Effects of relational peer victimization and social anxiety disorder on physiological and affective reactions to social exclusion. Front. Psychiatry.

[11] Rudolph KD, Davis MM, Skymba HV, Modi HH, Telzer EH (2021). Social experience calibrates neural sensitivity to social feedback during adolescence: A functional connectivity approach. Dev. Cognit. Neurosci..

[12] Ke T, De Simoni S, Barker E, Smith P (2022). The association between peer-victimisation and structural and functional brain outcomes: A systematic review. JCPP Adv..

[13] Theriot MT, Dulmus CN, Sowers KM, Johnson TK (2005). Factors relating to self-identification among bullying victims. Child. Youth Serv. Rev..

[14] Boyle KM, Clay-Warner J (2018). Shameful, “victims” and angry “survivors”: Emotion, mental health, and labeling sexual assault. Violence Vict..

[15] Weiss WM (2015). Community-based mental health treatments for survivors of torture and militant attacks in southern Iraq: A randomized control trial. BMC Psychiatry.

[16] Murray LK (2020). Effectiveness of the common elements treatment approach (CETA) in reducing intimate partner violence and hazardous alcohol use in Zambia (vatu): A randomized controlled trial. PLOS Med..

[17] Bosqui T (2023). What drives change in children receiving telephone-delivered common elements treatment approach (T-CETA)? A multiple n = 1 study with Syrian refugee children and adolescents in Lebanon. Child Abuse Neglect.

[18] Harkness KL, Hayden EP (2020). The Oxford Handbook of Stress and Mental Health.

[19] McCrory E, Ogle JR, Gerin MI, Viding E (2019). Neurocognitive adaptation and mental health vulnerability following maltreatment: The role of social functioning. Child Maltreat..

[20] Fortenbaugh FC (2017). Interpersonal early-life trauma alters amygdala connectivity and sustained attention performance. Brain Behav..

[21] Cisler JM (2017). Childhood trauma and functional connectivity between amygdala and medial prefrontal cortex: A dynamic functional connectivity and large-scale network perspective. Front. Syst. Neurosci..

[22] Ophuis RH, Olij BF, Polinder S, Haagsma JA (2018). Prevalence of post-traumatic stress disorder, acute stress disorder and depression following violence related injury treated at the emergency department: A systematic review. BMC Psychiatry.

[23] Seidel EM (2013). The impact of social exclusion vs. inclusion on subjective and hormonal reactions in females and males. Psychoneuroendocrinology.

[24] Wagels L (2017). Contextual exclusion processing: An fMRI study of rejection in a performance-related context. Brain Imaging Behav..

[25] Nietlisbach G, Maercker A (2009). Effects of social exclusion in trauma survivors with posttraumatic stress disorder. Psychol. Trauma.

[26] Williams KD, Cheung CKT, Choi W (2000). Cyberostracism: Effects of being ignored over the Internet. J. Pers. Soc. Psychol..

[27] Williams KD (2007). Ostracism. Annu. Rev. Psychol..

[28] Jobst A (2015). Effects of social exclusion on emotions and oxytocin and cortisol levels in patients with chronic depression. J. Psychiatr. Res..

[29] Jobst A (2014). Social exclusion leads to divergent changes of oxytocin levels in borderline patients and healthy subjects. Psychother. Psychosom..

[30] Zsila Á, Urbán R, Demetrovics Z (2018). Anger rumination and unjust world beliefs moderate the association between cyberbullying victimization and psychiatric symptoms. Psychiatry Res..

[31] Mwilambwe-Tshilobo L, Spreng RN (2021). Social exclusion reliably engages the default network: A meta-analysis of Cyberball. Neuroimage.

[32] Vijayakumar N, Cheng TW, Pfeifer JH (2017). Neural correlates of social exclusion across ages: A coordinate-based meta-analysis of functional MRI studies. Neuroimage.

[33] Versace A (2010). Abnormal left and right amygdala-orbitofrontal cortical functional connectivity to emotional faces: State versus trait vulnerability markers of depression in bipolar disorder. Biol. Psychiatry.

[34] Kumar P (2017). Increased neural response to social rejection in major depression. Depress Anxiety.

[35] McIver TA (2018). Peer victimization is associated with neural response to social exclusion. Merrill Palmer Q..

[36] McIver TA (2019). Functional connectivity across social inclusion and exclusion is related to peer victimization and depressive symptoms in young adults. J. Affect. Disord..

[37] Rudolph KD, Miernicki ME, Troop-Gordon W, Davis MM, Telzer EH (2016). Adding insult to injury: Neural sensitivity to social exclusion is associated with internalizing symptoms in chronically peer-victimized girls. Soc. Cogn. Affect. Neurosci..

[38] Radke S (2018). Immediate and delayed neuroendocrine responses to social exclusion in males and females. Psychoneuroendocrinology.

[39] Helpman L, Penso J, Zagoory-Sharon O, Feldman R, Gilboa-Schechtman E (2016). Endocrine and emotional response to exclusion among women and men; cortisol, salivary alpha amylase, and mood. Anxiety Stress Cop..

[40] Hartgerink CH, van Beest I, Wicherts JM, Williams KD (2015). The ordinal effects of ostracism: A meta-analysis of 120 Cyberball studies. PLOS ONE.

[41] Rajchert J, Konopka K, Oręziak H, Dziechciarska W (2022). Direct and displaced aggression after exclusion: Role of gender differences. J. Soc. Psychol..

[42] Oldfield RC (1971). The assessment and analysis of handedness: The Edinburgh inventory. Neuropsychologia.

[43] Ackenheil M, Stotz GG, Dietz-Bauer R, Vossen AR, Dietz R, Vossen-Wellmann A et al. Deutsche Fassung des Mini-International Neuropsychiatric Interview, (1999).

[44] Lehrl S, Triebig G, Fischer B (1995). Multiple choice vocabulary test MWT as a valid and short test to estimate premorbid intelligence. Acta Neurol. Scand..

[45] Reitan RM (1956). Trail Making Test. Manual for Administration, Scoring, and Interpretation.

[46] Aster v. M, Neubauer A, Horn R. (Hrsg). Wechsler Intelligenztest für Erwachsene. Deutschsprachige Bearbeitung und Adaptation des WAIS III von David Wechsler. Frankfurt/M.: Harcourt Test Services, (2006).

[47] World Medical Association (2013). World Medical Association Declaration of Helsinki: Ethical principles for medical research involving human subjects. JAMA.

[48] Krohne HW, Egloff B, Kohlmann CW, Tausch A (1996). Untersuchung mit einer deutschen Form der Positive and Negative Affect Schedule (PANAS). Diagnostica.

[49] van Beest I, Williams KD (2006). When inclusion costs and ostracism pays, ostracism still hurts. J. Personal. Soc. Psychol..

[50] Beck AT (2006). BDI-II Becks. Depressions Inventar.

[51] Laux L, Glanzmann P, Schaffner P (1982). The State-Trait Anxiety Inventory. Theoretical Basis and Manual. German Version.

[52] Franke GH (2000). Brief Symptom Inventory (BSI).

[53] Satow L. SCI. Stress- und Coping-Inventar. In Leibniz-Institut für Psychologie (ZPID) (Hrsg.), Open Test Archive. Trier: ZPID. (2012)

[54] Doering S (2007). Validierung der deutschen Version des Fragebogens zur Erfassung von DSM-IV Persönlichkeitsstörungen (ADP-IV). Z Psychosom. Med. Psychother.

[55] Abler B, Kessler H (2009). Emotion regulation questionnaire—Eine deutschsprachige Fassung des ERQ von Gross und John. Diagnostica.

[56] Marschner IC (2011). glm2: Fitting generalized linear models with convergence problems. R J..

[57] Stöcker T (2005). Automated quality assurance routines for fMRI data applied to a multicenter study. Hum. Brain Mapp..

[58] Ashburner J, Friston KJ (2005). Unified segmentation. Neuroimage.

[59] Whitfield-Gabrieli S, Nieto-Castanon A (2012). Conn: A functional connectivity toolbox for correlated and anticorrelated brain networks. Brain Connect..

[60] Fox MD, Raichle ME (2007). Spontaneous fluctuations in brain activity observed with functional magnetic resonance imaging. Nat. Rev. Neurosci..

[61] Lu P (2007). Global metabolic changes following loss of a feedback loop reveal dynamic steady states of the yeast metabolome. Metab. Eng..

[62] Cohen JS, Kendall PC (2015). Peer victimization among children and adolescents with anxiety disorders. Child Psychiatry Hum. Dev..

[63] Pontillo M (2019). Peer victimization and onset of social anxiety disorder in children and adolescents. Brain Sci..

[64] Vachon DD, Krueger RF, Rogosch FA, Cicchetti D (2015). Assessment of the harmful psychiatric and behavioral effects of different forms of child maltreatment. JAMA Psychiatry.

[65] Jankowski KF (2018). Feeling left out: Depressed adolescents may atypically recruit emotional salience and regulation networks during social exclusion. Soc. Cogn. Affect. Neurosci..

[66] Morawetz C, Bode S, Baudewig J, Heekeren HR (2017). Effective amygdala-prefrontal connectivity predicts individual differences in successful emotion regulation. Soc. Cogn. Affect. Neurosci..

[67] de Panfilis C, Riva P, Preti E, Cabrino C, Marchesi C (2015). When social inclusion is not enough: Implicit expectations of extreme inclusion in borderline personality disorder. Personal. Disord. Theor. Res. Treat..

[68] Schilbach L, Eickhoff SB, Rotarska-Jagiela A, Fink GR, Vogeley K (2008). Minds at rest? Social cognition as the default mode of cognizing and its putative relationship to the “default system” of the brain. Conscious Cogn..

[69] Buckner RL, Andrews-Hanna JR, Schacter DL (2008). The brain’s default network. Ann. N. Y. Acad. Sci..

[70] Aquino A (2020). Sense or sensibility? The neuro-functional basis of the structural matching effect in persuasion. Cognit. Affect. Behav. Neurosci..

[71] Li W, Mai X, Liu C (2014). The default mode network and social understanding of others: What do brain connectivity studies tell us. Front. Hum. Neurosci..

[72] Liston C (2014). Default mode network mechanisms of transcranial magnetic stimulation in depression. Biol. Psychiatry.

[73] Cavanna AE, Trimble MR (2006). The precuneus: A review of its functional anatomy and behavioural correlates. Brain.

[74] Weinbrecht A, Niedeggen M, Roepke S, Renneberg B (2018). Feeling excluded no matter what? Bias in the processing of social participation in borderline personality disorder. Neuroimage Clin..

[75] van Reemst L, Fischer TFC, Zwirs BWC (2016). Social Information Processing Mechanisms and Victimization. Trauma Violence Abuse.

[76] Domsalla M (2014). Cerebral processing of social rejection in patients with borderline personality disorder. Soc. Cogn. Affect. Neurosci..

[77] Kimble MO, Fleming K, Bennion KA (2013). Contributors to hypervigilance in a military and civilian sample. J. Interpers. Violence.

[78] Iffland B, Weitkämper A, Weitkämper NJ, Neuner F (2019). Attentional avoidance in peer victimized individuals with and without psychiatric disorders. BMC Psychol..

[79] Cha J (2016). Clinically anxious individuals show disrupted feedback between inferior frontal gyrus and prefrontal-limbic control circuit. J. Neurosci..

[80] Weik U, Maroof P, Zöller C, Deinzer R (2010). Pre-experience of social exclusion suppresses cortisol response to psychosocial stress in women but not in men. Horm. Behav..

