# Supplementary material for: Increased anger and stress and heightened connectivity between IFG and vmPFC in victims during social interaction
Source: Sci Rep. 2024 Apr 11;14:8471. doi: 10.1038/s41598-024-57585-y (PMC11009292; doi:10.1038/s41598-024-57585-y)
Supplement: Supplementary file 1 — Supplementary Information. [file 41598_2024_57585_MOESM1_ESM.pdf]

Supplements to Increased anger and stress and heightened connectivity between IFG and vmPFC in victims during social interaction

Severity Index

The development of a severity index (SI) was based on Severity of Violence Against Men Scales (SVAMS) and Severity of Violence Against Women Scales (SVAWS) by Marshall (1992 a, b). Several steps describe the procedure of the SI development: 1) conducting a semi-structured interview to collect information about a) violent types and b) modulatory weighting factors (duration, proximity to perpetrators and number of events). 2) a pre-rating of the violent types with the purpose of generating an externally determined violence index for each event. 3) creating a composite score (SI) using the violence index (a) and modulatory weighting factors (b).

Step1: Trained psychologists conducted semi-structured interviews in a sample of 138 individuals (78 females, 58 males) who had experienced any type of violence (a subsample of this group is part of the V group in the fMRI study presented in the manuscript). The interview guide underlying the interview is attached at the end of the supplements. Of the interviewed individuals, n=49 reported a single experience of violence, and n=86 reported repeated experiences. The maximal number of different violet types experienced was n=7. In total, 83 different events were extracted across all interviews; 18.1 % were coded as physical violence, 12.3 % as emotional violence, 3.6% as sexual violence and 2.0 % as material harm/deprivation.

Step 2: For the establishment of a violence index, all single violent events (n = 83) extracted from the semi-structured interviews were listed. In total, 100 individuals of which 50 were experts (medical doctors in psychiatry and psychotherapy; psychologists; psychotherapists) and 50 non-experts (other) evaluated the 80 events. The rating was performed according to five dimensions: *Aggressiveness*, *Vehemence*, *Threat*, *Seriousness* and *Abusiveness* on a scale from 1 (almost not at all) to 10 (very strong).

To evaluate the quality of the rating dimensions, we inspected reliability (Cronbach's α), item homogeneity (inter-item correlations), specificity, minimum and maximum values (see Supplementary Table 1). In addition, expert and non-expert groups were compared, finding a significant difference in the abuse dimension. Together, these results indicated that the abuse dimension was less qualified to characterize the events. Abuse may instead represent a violent type category itself. We therefore excluded this dimension in the further steps. Prior to determine the final composite score SI, the violence index for each event was composed by calculating mean values for each event across the four categories and evaluators. Afterwards,

these values were transformed by creating percentile ranks: VI = $\frac{Xn - Xmin}{(Xmax - Xmin)/10}$ (X_n = current mean of event n, X_{min} = minimal mean of all events, X_{max} = maximal mean of all events).

Supplementary Table 1: Quality check of the rating of violent events indicating minimum and maximum of inter item correlation, Cronbachs alpha for items (α) and standardised items (α *)

	Abusivness		Aggressiveness		Seriousness		Threat		Vehemence		VI	
	Min	Max	Min	Max	Min	Max	Min	Max	Min	Max	α	α
**event 1**	.50	.78	.58	.68	.58	.77	.65	.79	.66	.79	.89	.89
**event 2**	.44	.65	.66	.72	.66	.78	.70	.78	.63	.70	.90	.90
**event 3**	.53	.69	.68	.74	.66	.78	.70	.79	.66	.79	.91	.91
**event 4**	.43	.64	.53	.69	.53	.69	.53	.67	.62	.69	.85	.86
**event 5**	.67	.79	.77	.80	.77	.80	.77	.82	.77	.80	.93	.93
**event 6**	.67	.76	.74	.80	.74	.83	.77	.83	.75	.80	.93	.93
**event 7**	.36	.58	.54	.71	.49	.75	.48	.75	.48	.54	.86	.86
**event 8**	.65	.74	.71	.75	.71	.88	.75	.88	.74	.81	.93	.93
**event 9**	.78	.84	.82	.86	.80	.86	.85	.86	.80	.86	.95	.95
**event 10**	.68	.86	.75	.85	.75	.87	.77	.88	.79	.88	.94	.94
**event 11**	.22	.52	.57	.81	.53	.85	.60	.85	.53	.60	.89	.89
**event 12**	.28	.36	.77	.86	.75	.82	.82	.86	.75	.85	.94	.94
**event 13**	.46	.58	.74	.77	.75	.80	.74	.80	.75	.77	.92	.92
**event 14**	.60	.66	.79	.85	.79	.87	.81	.87	.82	.85	.95	.95
**event 15**	.49	.57	.69	.70	.70	.84	.69	.85	.70	.85	.92	.92
**event 16**	.74	.76	.86	.89	.88	.90	.87	.90	.86	.88	.96	.96
**event 17**	.59	.68	.72	.83	.72	.85	.77	.85	.79	.83	.93	.94
**event 18**	.67	.72	.81	.93	.80	.88	.87	.91	.80	.93	.96	.96
**event 19**	.44	.56	.75	.90	.84	.90	.75	.86	.77	.84	.94	.95
**event 20**	.58	.66	.74	.86	.74	.83	.77	.83	.80	.86	.94	.94
**event 21**	.74	.77	.79	.90	.82	.91	.80	.91	.79	.82	.95	.95
**event 22**	.32	.47	.45	.65	.45	.76	.46	.76	.54	.69	.84	.85
**event 23**	.44	.58	.28	.61	.28	.61	.61	.73	.49	.73	.82	.83
**event 24**	.34	.55	.54	.62	.62	.72	.61	.73	.54	.73	.86	.88
**event 25**	.26	.74	.23	.71	.18	.63	.37	.63	.18	.71	.74	.74
**event 26**	.66	.74	.73	.83	.74	.83	.74	.83	.74	.75	.92	.93
**event 27**	.32	.63	.54	.64	.43	.54	.51	.69	.43	.69	.83	.83
**event 28**	.33	.64	.69	.77	.57	.69	.62	.81	.57	.81	.90	.90
**event 29**	.54	.74	.56	.75	.49	.72	.64	.72	.49	.75	.88	.88
**event 30**	.55	.75	.61	.76	.69	.80	.61	.80	.62	.76	.89	.90
**event 31**	.62	.71	.75	.83	.78	.83	.77	.82	.75	.81	.94	.94
**event 32**	.64	.67	.77	.85	.70	.85	.77	.79	.70	.79	.93	.93
**event 33**	.47	.69	.47	.87	.45	.79	.60	.79	.45	.87	.87	.87
**event 34**	.53	.66	.58	.78	.58	.75	.71	.77	.62	.78	.90	.90
**event 35**	.48	.61	.71	.74	.64	.80	.74	.80	.64	.75	.91	.91

**event 36**	.56	.71	.72	.77	.65	.77	.70	.72	.65	.76	.91	.91
**event 37**	.52	.66	.64	.71	.57	.85	.68	.85	.57	.71	.89	.90
**event 38**	.49	.68	.62	.72	.49	.74	.70	.74	.49	.72	.89	.88
**event 39**	.49	.69	.47	.76	.43	.68	.61	.68	.43	.76	.86	.86
**event 40**	.45	.77	.59	.76	.44	.81	.61	.81	.44	.76	.88	.88
**event 41**	.52	.67	.79	.81	.72	.88	.81	.88	.72	.81	.94	.94
**event 42**	.51	.72	.62	.74	.59	.84	.56	.84	.56	.74	.87	.89
**event 43**	.28	.42	.35	.81	.34	.80	.62	.80	.34	.81	.84	.85
**event 44**	.47	.70	.68	.74	.72	.79	.68	.79	.74	.77	.90	.92
**event 45**	.41	.60	.60	.80	.63	.76	.60	.73	.73	.80	.89	.90
**event 46**	.52	.72	.62	.78	.72	.82	.62	.80	.78	.82	.91	.92
**event 47**	.70	.82	.77	.87	.76	.77	.76	.84	.77	.87	.94	.94
**event 48**	.53	.67	.75	.80	.73	.92	.73	.92	.73	.80	.93	.93
**event 49**	.68	.71	.79	.84	.82	.84	.79	.85	.83	.85	.94	.95
**event 51**	.56	.73	.70	.87	.78	.88	.70	.88	.76	.88	.93	.94
**event 52**	.66	.76	.74	.89	.74	.82	.80	.86	.82	.89	.94	.94
**event 53**	.76	.84	.80	.90	.80	.86	.86	.93	.85	.93	.96	.96
**event 54**	.66	.70	.81	.87	.86	.90	.81	.90	.82	.88	.96	.96
**event 55**	.28	.53	.65	.76	.64	.85	.65	.85	.64	.74	.89	.91
**event 56**	.73	.78	.88	.91	.88	.95	.88	.95	.88	.91	.97	.97
**event 57**	.51	.63	.81	.86	.76	.90	.85	.90	.76	.86	.95	.95
**event 58**	.77	.78	.83	.91	.85	.94	.83	.94	.85	.91	.96	.96
**event 60**	.52	.64	.73	.85	.84	.86	.73	.84	.81	.86	.94	.95
**event 61**	.34	.39	.23	.74	.31	.64	.23	.64	.33	.74	.68	.76
**event 63**	.54	.64	.79	.81	.71	.83	.75	.83	.71	.80	.93	.93
**event 64**	.72	.81	.75	.90	.75	.89	.83	.89	.81	.90	.95	.95
**event 65**	.58	.72	.52	.80	.52	.71	.59	.74	.57	.80	.86	.88
**event 66**	.35	.51	.56	.74	.51	.66	.60	.69	.51	.74	.85	.87
**event 67**	.60	.65	.74	.83	.76	.88	.72	.88	.72	.76	.93	.94
**event 68**	.61	.74	.75	.81	.79	.89	.77	.89	.75	.85	.94	.94
**event 69**	.58	.68	.73	.82	.68	.82	.73	.80	.68	.76	.92	.92
**event 70**	.71	.79	.82	.88	.76	.88	.84	.87	.76	.87	.95	.95
**event 71**	.58	.71	.67	.84	.60	.80	.68	.80	.60	.84	.90	.91
**event 72**	.62	.69	.81	.86	.74	.86	.79	.81	.74	.81	.94	.94
**event 73**	.61	.67	.75	.77	.64	.75	.70	.78	.64	.78	.91	.91
**event 74**	.63	.72	.71	.85	.66	.76	.75	.76	.66	.85	.92	.92
**event 75**	.35	.64	.58	.75	.58	.75	.42	.60	.42	.68	.84	.86
**event 76**	.45	.56	.51	.62	.39	.74	.50	.74	.39	.62	.83	.83
**event 77**	.41	.57	.66	.75	.64	.80	.68	.80	.64	.72	.90	.90
**event 78**	.63	.70	.64	.71	.55	.71	.71	.71	.55	.71	.89	.89
**event 79**	.55	.63	.77	.81	.79	.88	.77	.88	.81	.88	.95	.95
**event 80**	.43	.61	.63	.69	.63	.78	.63	.78	.63	.76	.90	.90
**event 81**	.43	.57	.63	.68	.54	.76	.65	.76	.54	.68	.88	.88
**event 82**	.43	.55	.48	.71	.48	.70	.61	.80	.65	.80	.87	.88
**event 83**	.41	.62	.57	.74	.57	.80	.58	.80	.60	.74	.87	.88

Supplementary Table 2: Group comparison of the rating dimension mean scores

Scale	experts	non-experts	*t* (98)	*p*
**Abusivness**	6.50	7.15	-2.109	.037*
**Aggressivness**	6.73	7.12	-1.447	.151
**Seriousness**	7.43	7.70	-1.197	.234
**Threat**	7.11	7.42	-1.326	.188
**Vehemence**	6.59	6.99	-1.506	.135

*p < .05

The coefficients of the interrater reliabilities (Kendall's Tau τb) of the five severity categories for experts and non-experts vary significantly between τb = .85 and τb = .90 (p = .00). However, despite of the variation they are to be evaluated as good.

Step 3: For the final generation of SI information about modulatory weighting factors three independent experimenters assigned a violence index to each situation that was indicated by a participant. Furthermore, the experimenters used the respective event description, to estimate the weight of the influence factors. The instruction for the weighting factors was the following: Binary code for the perpetrator differentiating close persons (1) and others (0); a categorical code concerning the duration with a single experience (1), a repeating duration of < 1 year (2) or a duration =/> 1 year (chronic) (3).

For each event, a sum is calculated of the violence index and the proximity to the perpetrator, while the result of adding both factors is multiplied by the duration of the experience. Violence by a caregiver could increase the severity, whereas violence by a strange perpetrator had no effects. The duration factor is multiplied, as this ensures that the three weightings have significantly different effects. In case a victim was exposed to the same type of violence several times, this was considered by adding up these events accordingly. The final composition of a global SI for each victim was thus calculated by the following formula:

$$SI = [(VI_{e(1)} + P_{e(1)}) *D_{e(1)}] + [(VI_{e(2)} + P_{e(2)})*D_{e(2)}] + \dots [(VI_{e(n)} + P_{e(n)}) *D_{e(n)}]$$

**VI = violent index, P = closeness to the perpetrator, D = duration of the experience, e(n) = event n.*

To test the correspondence of the classification of the full three rating results (ordinal data) we determined interrater reliability using Krippendorff's Alpha (KALPHA, using SPSS custom dialog of Hayes and Krippendorff, 2007). With $\alpha = .97$, we found a high interrater

reliability (Hayes and Krippendorff, 2007). An overview on the ratings is given in Supplementary Table 3.

Supplementary Table 3: Final mean SI scores including the scores of each independent rating depicted for each participant

participant	Rater 1	Rater 2	Rater 3	SI
**1**	117	102	117	112
**2**	49	61	61	57
**3**	15	15	10	13
**4**	60	51	69	60
**5**	39	33	39	37
**6**	67	80	77	75
**7**	84	63	84	77
**8**	18	14	18	17
**9**	39	39	39	39
**10**	42	42	42	42
**11**	36	42	42	40
**12**	42	51	48	47
**13**	20	20	16	19
**14**	26	26	29	27
**15**	84	59	81	75
**16**	6	6	6	6
**17**	45	39	39	41
**18**	109	107	86	101
**19**	72	48	60	60
**20**	89	92	83	88
**21**	9	9	9	9
**22**	24	21	30	25
**23**	18	18	18	18
**24**	22	18	24	21
**25**	100	94	110	101
**26**	93	93	96	94
**27**	25	26	30	27
**28**	11	11	21	14
**29**	9	9	10	9
**30**	42	42	42	42
**31**	10	10	10	10
**32**	30	16	27	24
**33**	50	50	50	50
**34**	12	9	11	11
**35**	51	51	51	51
**36**	25	21	33	26
**37**	99	90	99	96

**38**	75	73	70	73
**39**	12	12	12	12
**40**	30	30	33	31
**41**	72	72	66	70
**42**	42	45	39	42
**43**	68	62	62	64
**44**	36	36	39	37
**45**	12	12	12	12
**46**	195	192	195	194
**47**	18	18	18	18
**48**	90	81	96	89
**49**	51	48	51	50
**50**	42	38	47	42
**51**	30	30	30	30
**52**	9	9	9	9
**53**	52	52	52	52
**54**	54	54	60	56
**55**	30	30	30	30
**56**	36	48	39	41
**57**	42	48	36	42
**58**	54	60	63	59
**59**	23	23	23	23
**60**	63	66	63	64
**61**	119	113	128	120
**62**	89	104	76	90
**63**	30	30	30	30
**64**	19	19	19	19
**65**	50	62	77	63
**66**	69	72	81	74
**67**	34	40	40	38
**68**	42	42	42	42
**69**	66	75	93	78
**70**	30	30	30	30
**71**	48	51	57	52
**72**	96	90	96	94
**73**	84	84	87	85
**74**	30	30	30	30
**75**	42	36	51	43
**76**	42	42	44	43
**77**	57	51	57	55
**78**	9	9	9	9
**79**	49	49	58	52
**80**	126	132	174	144
**81**	10	9	10	10
**82**	39	42	42	41

**83**	9	16	15	13
**84**	42	42	48	44
**85**	51	51	51	51
**86**	103	103	115	107
**87**	19	60	42	40
**88**	60	60	60	60
**89**	30	36	36	34
**90**	53	99	99	84
**91**	30	30	30	30
**92**	57	48	57	54
**93**	10	9	10	10
**94**	10	10	10	10
**95**	12	12	12	12
**96**	104	104	104	104
**97**	9	9	9	9
**98**	30	30	30	30
**99**	52	90	75	72
**100**	81	72	78	77
**101**	123	117	126	122
**102**	20	24	24	23
**103**	48	48	48	48
**104**	73	70	73	72
**105**	9	9	9	9
**106**	25	25	25	25
**107**	27	27	24	26
**108**	16	15	15	15
**109**	14	36	14	21
**110**	72	75	75	74
**111**	10	9	9	9
**112**	69	75	75	73
**113**	46	51	60	52
**114**	49	50	49	49
**115**	55	54	55	55
**116**	9	9	9	9
**117**	60	60	45	55
**118**	40	41	41	41
**119**	9	3	9	7
**120**	39	39	38	39
**121**	30	30	30	30
**122**	36	36	42	38
**123**	12	8	9	10
**124**	105	105	105	105
**125**	57	57	57	57
**126**	12	12	12	12
**127**	24	42	39	35

**128**	27	27	27	27
**129**	83	90	70	81
**130**	59	55	59	58
**131**	19	16	22	19
**132**	51	54	54	53
**133**	9	9	12	10
**134**	69	75	87	77
**135**	62	63	57	61
**136**	132	135	135	134
**137**	48	48	48	48

References

Hayes, A. F., & Krippendorff, K. (2007). Answering the Call for a Standard Reliability Measure for Coding Data. *Communication Methods and Measures*, *1*(1), 77–89. http://doi.org/10.1080/19312450709336664

Marshall, L. L. (1992a). Development of the severity of violence against women scales. *Journal of Family Violence*, *7*(2), 103–121. http://doi.org/10.1007/BF00978700

Marshall, L. L. (1992b). The Severity of Violence Against Men Scales. *Journal of Family Violence*, *7*(3), 189–203. http://doi.org/10.1007/BF00979027

Procedure

Each block lasted about 50 seconds. In FG and PG blocks participants allegedly played Cyberball with their teammates receiving the ball in 60% of all cases when they were in the inclusion condition. The difference between the FG and PG was the game context: In FG runs participants could play at their self-chosen speed. In contrast, participants were informed that reaction time would be essential for a good group performance in PG runs. For faster reactions, the group would earn more points. Relatively slow reactions would be indicated by a red frame encircling the corresponding virtual figure. The warning frame appeared in 20% of the throws of the teammates, whereas it appeared in about 50% of all throws for the participant. To improve credibility, the reaction time of the virtual figures was flexible, calculated from the speed of the

first 10 seconds of each block. Thereby, it was ensured that the virtual figures representing the teammates were reacting faster than the participant providing a potential logical reason for the following exclusion.

The order of inclusion and exclusion blocks (3 respectively) within FG or PG was randomized except for the paradigm start, which was always an inclusion condition. All PG blocks were presented in one run. O blocks were inserted between FG blocks.

Pathological symptoms were assessed either within the same session or at a separate appointment in case the participant was exhausted.

Credibility

To ensure credibility issues did not interfere with our manipulation, we employed a special questionnaire to check whether the cover story was believed by the individual participant or not. Believing the task or not did not influence anger ($F = .002, p =.966$) nor stress ratings ($F = .139, p = .710$).

Supplementary table 4

Anatomical Region	x	y	z	N studies	Volume (mm3)	cluster
R sgACC	4	32	-6	17	5448	1
L vmPFC	-10	44	-10			
R vmPFC	4	44	-12			
L IFG	-46	32	-10	8	1992	2
L Lateral OFC	-38	32	-12			
L Posterior Cingulate	-8	-56	12	7	1344	3
R Precuneus	4	-40	40	6	1000	4
R Precentral Gyrus	54	-8	4	4	736	5

Table 4. *Anatomical labeling and MNI coordinates from Vijayakumar et al., 2017 using 29 studies and N = 857.*
Notes. L = left hemisphere, R = right hemisphere. Only grey matter is included in table. SgACC = subgenual Anterior Cingulate Cortex, vmPFC = Ventromedial Prefrontal Cortex, IFG = Inferior Frontal Gyrus, OFC = Orbitofrontal Cortex

Supplementary table 5

	Df	Res. Deviance	DF	Res. Deviance	*F*	*p*
Anger						
Condition	1	3.92	278	112.36	11.65	<.001
Context	1	2.22	277	110.14	6.6	.01*
Group	1	5.9	276	104.24	17.53	<.001
Gender	1	2.96	274	101.17	8.81	.003
Task Credibility	1	.11	275	104.13	.34	.56
Group x Gender	1	.61	273	100.55	1.82	.18
Condition x Context	1	.44	272	100.11	1.32	.25
Condition x Group	1	.04	271	100.07	.11	.74
Context x Group	1	.46	270	99.61	1.38	.24
Condition x Context x Group	1	.04	269	99.57	.12	.73
Stress						
Condition	1	.014	278	110.96	.042	.839
Context	1	4.25	277	106.71	12.97	<.001
Group	1	3.72	276	102.99	11.36	<.001
Gender	1	2.01	274	100.98	6.14	.014
Task Credibility	1	.001	275	102.99	.002	.97
Group x Gender	1	1.26	273	99.71	3.86	.051
Condition x Context	1	.21	272	99.5	.65	.42
Condition x Group	1	.02	271	99.48	.05	.82
Context x Group	1	.49	270	99.99	1.51	.22
Condition x Context x Group	1	.20	269	99.79	.62	.43

Teammates						
Condition	1	.54	278	22.23	8.09	.005
Context	1	.02	277	22.21	.27	.60
Group	1	.002	276	22.21	.02	.88
Gender	1	.58	274	20.81	8.67	<.004
Task Credibility	1	.81	275	21.39	12.17	<.001
Group x Gender	1	.03	273	20.78	.46	.50
Condition x Context	1	.00	272	20.78	.001	.98
Condition x Group	1	.03	271	20.75	.40	.53
Context x Group	1	.01	270	20.74	.15	.70
Condition x Context x Group	1	.002	269	20.74	.04	.85

Table 5. *Main and interaction effects of the Generalized Linear Model for participants' ratings of anger, stress and teammates.*

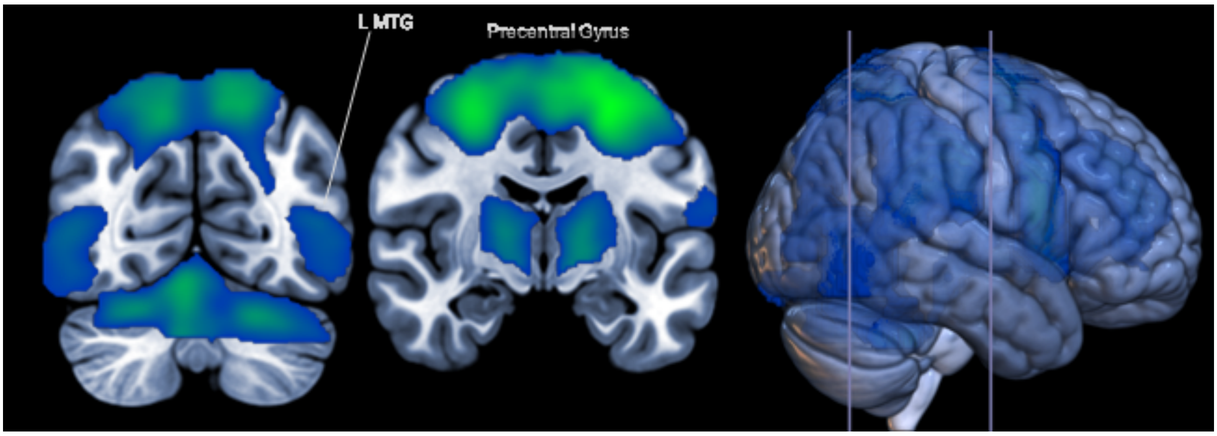

Supplementary Figure 1. Multi slice images of the BOLD signal t-contrast of Victims (N = 39) over Non-Victims (N = 33) in IN > EX during the Cyberball Game

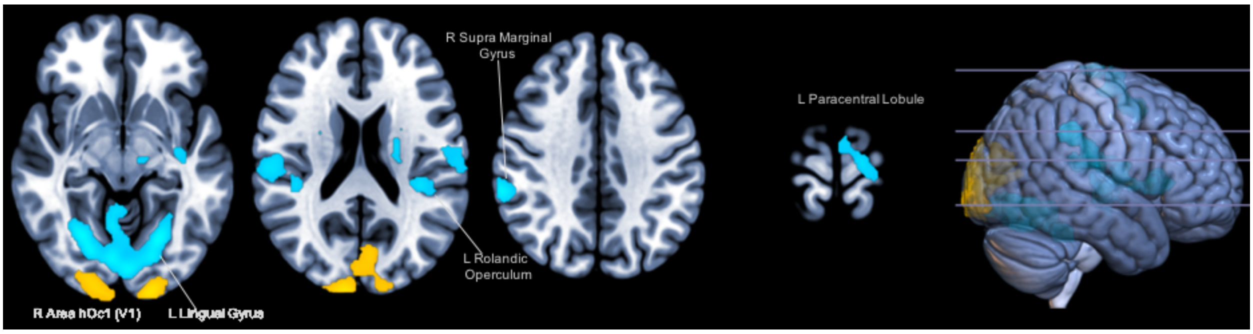

Supplementary Figure 2. Multi slice images for the BOLD signal F-contrast of context (FG vs. PG, yellow) during the Cyberball Game

Supplementary Table 6, MNI coordinates of peak voxel for the t-contrast of IN > EX

Anatomical Region	x	y	z	t	k	cluster
L Precentral Gyrus	-26	-10	56	21.33	62819	1
L Middle Temporal Gyrus	-48	-66	10	7.83	1714	2
N/A	50	-28	-12	4.79	4	3
N/A	34	-16	-8	4.82	3	4
L Lingual Gyrus	-2	-68	2	4.82	2	5

Note. L = left hemisphere, R = right hemisphere. Only grey matter is included in table.

Supplementary Table 7, MNI coordinates of peak voxel for the t-contrast of FG >. PG

Anatomical Region	x	y	z	t	k	cluster
R Area hOc1 (V1)	14	-104	10	6.91	2716	1

Note. R = right hemisphere. Only grey matter is included in table.

Supplementary Table 8, MNI coordinates of peak voxel for the F-contrast of PG > FG

Anatomical Region	x	y	z	t	k	cluster
L Lingual Gyrus	-22	-68	-8	5.62	2966	1
L Paracentral Lobule	-16	-20	76	4.13	688	4
R Supra Marginal Gyrus	62	-38	38	5.27	678	5
L Rolandic Operculum	-38	-34	20	4.40	355	6

Note. L = left hemisphere, R = right hemisphere. Only grey matter is included in table.

Interviewleitfaden IuK

Durchgeführt von:	Datum:	Code:

1. Studienhintergrund und -einwilligung

- ❑ Studienhintergrund, Inhalt der Teilnahme, Dauer, Aufwandsentschädigung
- ❑ Rechtliche Aufklärung, Datenschutz, Schweigepflicht
- ❑ Einwilligung unterschrieben
- ❑ Kopie erwünscht und ausgehändigt

2. Demographie

- Geburtsdatum/-jahr ______________________
- Geburtsland ______________________
- Familienstand ______________________
- Kinder ______________________
- Wohnverhältnisse: Mit wem leben Sie zusammen? ______________________
- Höchster Bildungsabschluss: ❑ Hauptschule/ Volksschule ❑ Realschule/ mittlere Reife ❑ Fachabitur ❑ Abitur ❑ Studium ❑ kein Abschluss

3. Wie häufig waren Sie in den letzten zwei Jahren in ärztlicher Behandlung?

ambulant: ca. ___ Mal   stationär: ca. ___ Mal

4. War der Grund der Arztbesuche

- ❑ immer der gleiche
- ❑ verschiedene Gründe
- ❑ Vorsorge *(z. B. Gynäkologie, Rezept abholen, Zahnarzt, Impfung)*

5. Haben Sie jemals unter den folgenden Erkrankungen oder Störungen gelitten?

- ❑ Verletzungen, funktionelle Beeinträchtigung, dauerhafte Behinderung
- ❑ chronische Schmerzsyndrome, Harnwegsinfekte, Magen-Darm-Erkrankungen
- ❑ Atemwegserkrankungen *(z. B. Asthma)*
- ❑ Hauterkrankungen *(z. B. Neurodermitis)*
- ❑ Schlafstörungen
- ❑ Depressionen
- ❑ Essstörungen
- ❑ Suizidalität/ lebensmüde Gedanken
- ❑ posttraumatische Belastungsreaktionen durch bestimmtes Ereignis
- ❑ geschlechtsspezifische Erkrankungen *(z. B. Brust, Gebärmutter, Prostata)*
- ❑ Sonstiges (auch Operationen, Allergien) ______________________

6. Überblick Gewalterfahrungen allgemein: Waren Sie jemals Opfer
(auf die Einzelheiten wird dann im weiteren Gesprächsverlauf eingegangen, Zutreffendes ankreuzen)

- ❑ körperlicher Gewalt (jede Form tätlicher Angriffe, z.B. schlagen, schütteln, treten, anspucken)
- ❑ psychischer Gewalt (z.B. ständige Kritik, Erniedrigung, Beleidigung, Mobbing, Stalking/Verfolgung)
- ❑ sexueller Gewalt (z.B. Vornahme sexueller Handlungen ohne Ihr Einverständnis, Nötigung, Vergewaltigung, sexuelle Belästigung)
- ❑ wirtschaftlicher Gewalt (z.B. Diebstahl von Besitz oder Geld, Zurückhalten des Einkommens, Entzug von Grundbedürfnissen)

A. Körperliche Gewalt

Sie haben vorhin angegeben, dass sie Opfer körperlicher Gewalt geworden sind. Können Sie dies etwas genauer beschreiben, bevor ich noch einige konkretere Fragen dazu stelle?

____

____

____

1. **Alter: Wie alt waren Sie?** ____________________
2. **Über welchen Zeitraum ist das passiert?** ____________________
3. **Wie häufig ist das vorgekommen?** ____________________
4. **Ort: Wo hat die Gewalterfahrung stattgefunden?**

❑ Arbeit
❑ zu Hause
❑ In der Stadt
❑ unterwegs
❑ in der Schule
❑ ____________________

5. **Täter: Wer hat die Gewalt ausgeübt?**

❑ Partner(in)/ ehemalige(r) Partner(in)
❑ Familie
❑ Freunde/ Bekannte
❑ ____________________
❑ Fremde
❑ Institutionen wie Polizei, Ordnungsamt, Arbeitsagentur
❑ berufliches Umfeld: Vorgesetzte, Kollegen

6. **Verhältnis: In welchem Verhältnis standen Sie zum Täter/zur Täterin?**

❑ Arbeitgeber
❑ Vorgesetzte
❑ Kollegen
❑ ____________________
❑ Familie
❑ Partner/ ehemalige(r) Partner(in)
❑ Freunde/ Bekannte
❑ Fremde

7. **Abhängigkeit: Waren Sie auf irgendeine Art an diese Person gebunden?**

❑ emotional
❑ sozial
❑ wirtschaftlich
❑ ____________________

8. **Folgen: Gab es …….. Probleme/Verletzungen aufgrund dieser Gewalterfahrung?**

❑ körperliche (Verletzungen, Krankheiten, dauerhafte/funktionelle Beeinträchtigungen)
Wenn ja: welche? ____________________
❑ psychische (Angst, Scham, Schuldgefühle, Rückzug, Wut)
Wenn ja: welche? ____________________
❑ wirtschaftliche (Geldverlust, Verlust der Wohnung)
Wenn ja: welche? ____________________
❑ soziale (sozialer Rückzug, Aufgabe von Freizeitaktivitäten, Spannungen im Familien- oder Freundeskreis)
Wenn ja: welche? ____________________
❑ berufliche (Verlust der Arbeit, unfaire Behandlung am Arbeitsplatz, häufiges Fehlen/erhöhte Fehlzeiten)
Wenn ja: welche? ____________________

9. **Wie haben Sie reagiert?**

____

____

____

a. Abwehr/ Gegenwehr? ____________________
b. Was ist danach passiert? ____________________
c. Hat sich Ihre Situation danach verbessert oder verschlechtert? ❑ besser ❑ schlechter ❑ keine Änderung
d. Würden Sie erneut so handeln? ❑ ja ❑ nein
Sondern: ____________________

Falls keine Gegenwehr/Reaktion:

e. Können Sie mir einen Grund dafür nennen? ____

f. Haben dabei Gefühle wie z.B. Angst, Scham, Hilflosigkeit eine Rolle gespielt?

____

g. Hat sich Ihre Situation danach verbessert oder verschlechtert? ❑ besser ❑ schlechter ❑ keine Änderung

h. Würden Sie erneut so handeln? ❑ ja ❑ nein

15. Als wie schwer haben Sie das Erlebte *damals* eingeschätzt? Skala 0 bis 10 ____

Als wie schwer schätzen Sie es *heute* ein? Skala 0 bis 10 ____

11. Sind Sie in Folge dieser Erfahrung in irgendeiner Art behandelt worden? ❑ ja ❑ nein

Falls ja: Wo? ❑ Krankenhaus ❑ Therapeut ❑ Hausarzt ❑ _______________________

10. Haben Sie jemandem von diesen Vorfällen/Ereignissen erzählt? ❑ ja ❑ nein

Falls ja: a) Wem haben Sie sich anvertraut?

❑ niemandem ❑ Hausarzt/in , Krankenhausarzt ❑ Beratungsstelle
❑ Familie ❑ sonstiges medizinisches Fachpersonal ❑ Opferhilfsorganisation
❑ Freunden ❑ Polizei
❑ ____________________

b) Wieso dieser Person/Institution? ____

c) Wann haben Sie sich dieser Person/Organisation anvertraut? ___________________________

d) War das Reden hilfreich? ❑ ja ❑ nein

Können Sie das etwas genauer erklären?

____

____

12. Haben Sie wegen dieser Erfahrung jemals professionelle Hilfe aufgesucht? ❑ ja ❑ nein

Falls ja: a) Wo? ❑ Beratungsstelle ❑ Hilfsorganisation ❑ Kirche/Gemeinde ❑ Psychotherapeut ❑ Justiz (Polizei, Gericht, SA) ❑ _______________________

b) Wie lange waren Sie dort in „Beratung“? _________________________________

c) Woher kam der Kontakt/die Information? _______________________________________

d) Wie lange hat es gedauert, bis Sie Hilfe gesucht haben? ______________________________

e) Hat Ihnen die Beratung dort geholfen? ❑ ja ❑ nein

Falls ja: Können Sie das etwas genauer erklären? ____________________________________

Falls nein: Was hatten Sie sich anders vorgestellt/erhofft? ______________________________

Falls nein: Warum nicht? Was haben Sie befürchtet? ____

13. Was hat Ihnen am ehesten geholfen, diese Erlebnisse zu verarbeiten?

❑ Freunde (Gespräche, Nähe) ❑ professionelle Hilfe
❑ Familie (Gespräche, Nähe) ❑ vergangene Zeit
❑ ____

14. Glauben Sie, dass es Gründe gibt, dass Ihnen dieses Ereignis zugestoßen ist? ❑ ja ❑ nein

Falls ja: Woran machen Sie das fest? ____

____

B. Psychische Gewalt

Sie haben vorhin angegeben, dass sie Opfer psychischer Gewalt geworden sind. Können Sie dies etwas genauer beschreiben, bevor ich noch einige konkretere Fragen dazu stelle?

____

____

____

1. **Alter: Wie alt waren Sie?** ____________________
2. **Über welchen Zeitraum ist das passiert?** ____________________
3. **Wie häufig ist das vorgekommen?** ____________________
4. **Ort: Wo hat die Gewalterfahrung stattgefunden?**
   - ❑ Arbeit
   - ❑ zu Hause
   - ❑ In der Stadt
   - ❑ unterwegs
   - ❑ in der Schule
   - ❑ ____________________
5. **Täter: Wer hat die Gewalt ausgeübt?**
   - ❑ Partner(in)/ ehemalige(r) Partner(in)
   - ❑ Familie
   - ❑ Bekannte
   - ❑ ____________________
   - ❑ Fremde
   - ❑ Institutionen wie Polizei, Ordnungsamt, Arbeitsagentur
   - ❑ berufliches Umfeld: Vorgesetzte, Kollegen
6. **Verhältnis: In welchem Verhältnis standen Sie zum Täter/zur Täterin?**
   - ❑ Arbeitgeber
   - ❑ Vorgesetzte
   - ❑ Kollegen
   - ❑ ____________________
   - ❑ Familie
   - ❑ Partner(in) ehemalige(r) Partner(in)
   - ❑ Freunde/ Bekannte
   - ❑ Fremde
7. **Abhängigkeit: Waren Sie auf irgendeine Art an diese Person gebunden?**
   - ❑ emotional
   - ❑ sozial
   - ❑ wirtschaftlich
   - ❑ ____________________
8. **Folgen: Gabe es .... Probleme/Verletzungen aufgrund dieser Gewalterfahrung?**
   - ❑ körperliche (Verletzungen, Krankheiten, dauerhafte/funktionelle Beeinträchtigungen)
     Wenn ja: welche? (Dokublatt Hessen beilegen!) ____________________
   - ❑ psychische (Angst, Scham, Schuldgefühle, Rückzug, Wut)
     Wenn ja: welche? ____________________
   - ❑ wirtschaftliche (Geldverlust, Verlust der Wohnung)
     Wenn ja: welche? ____________________
   - ❑ soziale (sozialer Rückzug, Aufgabe von Freizeitaktivitäten, Spannungen im Familien- oder Freundeskreis)
     Wenn ja: welche? ____________________
   - ❑ berufliche (Verlust der Arbeit, unfaire Behandlung am Arbeitsplatz, häufiges Fehlen/erhöhte Fehlzeiten)
     Wenn ja: welche? ____________________
9. **Wie haben Sie reagiert?**

____

____

____

a. Abwehr/ Gegenwehr? ____________________

b. Was ist danach passiert? ____________________

c. Hat sich Ihre Situation danach verbessert oder verschlechtert? ❑ besser ❑ schlechter ❑ keine Änderung

d. Würden Sie erneut so handeln? ❑ ja ❑ nein

Sondern: ____________________

Falls keine Gegenwehr/Reaktion:

e. Können Sie mir einen Grund dafür nennen? ____

f. Haben dabei Gefühle wie z.B. Angst, Scham, Hilflosigkeit eine Rolle gespielt?

____

g. Hat sich Ihre Situation danach verbessert oder verschlechtert? ❑ besser ❑ schlechter ❑ keine Änderung

h. Würden Sie erneut so handeln? ❑ ja ❑ nein

15. Als wie schwer haben Sie das Erlebte *damals* eingeschätzt? Skala 0 bis 10 ____

Als wie schwer schätzen Sie es *heute* ein? Skala 0 bis 10 ____

11. Sind Sie in Folge dieser Erfahrung in irgendeiner Art behandelt worden? ❑ ja ❑ nein

Falls ja: Wo? ❑ Krankenhaus ❑ Therapeut ❑ Hausarzt ❑ ________________________

10. Haben Sie jemandem von diesen Vorfällen/Ereignissen erzählt? ❑ ja ❑ nein

Falls ja: a) Wem haben Sie sich anvertraut?

❑ niemandem ❑ Hausarzt/in , Krankenhausarzt ❑ Beratungsstelle
❑ Familie ❑ sonstiges medizinisches Fachpersonal ❑ Opferhilfsorganisation
❑ Freunden ❑ Polizei
❑ ____________________

b) Wieso dieser Person/Institution? ____

c) Wann haben Sie sich dieser Person/Organisation anvertraut? ____________________________

d) War das Reden hilfreich? ❑ ja ❑ nein

Können Sie das etwas genauer erklären?

____

____

12. Haben Sie wegen dieser Erfahrung jemals professionelle Hilfe aufgesucht? ❑ ja ❑ nein

Falls ja: a) Wo? ❑ Beratungsstelle ❑ Hilfsorganisation ❑ Kirche/Gemeinde ❑ Psychotherapeut ❑ Justiz (Polizei, Gericht, SA) ❑ ________________________

b) Wie lange waren Sie dort in „Beratung“? ___________________________________

c) Woher kam der Kontakt/die Information? ______________________________________

d) Wie lange hat es gedauert, bis Sie Hilfe gesucht haben? ________________________________

e) Hat Ihnen die Beratung dort geholfen? ❑ ja ❑ nein

Falls ja: Können Sie das etwas genauer erklären? ______________________________________

Falls nein: Was hatten Sie sich anders vorgestellt/erhofft? _______________________________

Falls nein: Warum nicht? Was haben Sie befürchtet? ____

13. Was hat Ihnen am ehesten geholfen, diese Erlebnisse zu verarbeiten?

❑ Freunde (Gespräche, Nähe) ❑ professionelle Hilfe
❑ Familie (Gespräche, Nähe) ❑ vergangene Zeit
❑ ____

14. Glauben Sie, dass es Gründe gibt, dass Ihnen dieses Ereignis zugestoßen ist? ❑ ja ❑ nein

Falls ja: Woran machen Sie das fest? ____

____

C. Sexuelle Gewalt

Sie haben vorhin angegeben, dass sie Opfer sexueller Gewalt geworden sind. Können Sie dies etwas genauer beschreiben, bevor ich noch einige konkretere Fragen dazu stelle?

____

____

____

1. **Alter: Wie alt waren Sie?** __________________________

2. **Über welchen Zeitraum ist das passiert?** __________________________

3. **Wie häufig ist das vorgekommen?** __________________________

4. **Ort: Wo hat die Gewalterfahrung stattgefunden?**
   - ❑ Arbeit
   - ❑ zu Hause
   - ❑ In der Stadt
   - ❑ unterwegs
   - ❑ in der Schule
   - ❑ ________________________

5. **Täter: Wer hat die Gewalt ausgeübt?**
   - ❑ Partner(in)/ ehemalige(r) Partner(in)
   - ❑ Familie
   - ❑ Bekannte
   - ❑ ________________________
   - ❑ Fremde
   - ❑ Institutionen wie Polizei, Ordnungsamt, Arbeitsagentur
   - ❑ berufliches Umfeld: Vorgesetzte, Kollegen

6. **Verhältnis: In welchem Verhältnis standen Sie zum Täter/zur Täterin?**
   - ❑ Arbeitgeber
   - ❑ Vorgesetzte
   - ❑ Kollegen
   - ❑ ________________________
   - ❑ Familie
   - ❑ Partner(in)/ ehemalige(r) Partner(in)
   - ❑ Freunde/ Bekannte
   - ❑ Fremde

7. **Abhängigkeit: Waren Sie auf irgendeine Art an diese Person gebunden?**
   - ❑ emotional
   - ❑ sozial
   - ❑ wirtschaftlich
   - ❑ ________________________

8. **Folgen: Gabe es …….. Probleme/Verletzungen aufgrund dieser Gewalterfahrung?**
   - ❑ körperliche (Verletzungen, Krankheiten, dauerhafte/funktionelle Beeinträchtigungen)
     Wenn ja: welche? (Dokublatt Hessen beilegen!) __________________________________
   - ❑ psychische (Angst, Scham, Schuldgefühle, Rückzug, Wut)
     Wenn ja: welche? ____
   - ❑ wirtschaftliche (Geldverlust, Verlust der Wohnung)
     Wenn ja: welche? ____
   - ❑ soziale (sozialer Rückzug, Aufgabe von Freizeitaktivitäten, Spannungen im Familien- oder Freundeskreis)
     Wenn ja: welche? ____
   - ❑ berufliche (Verlust der Arbeit, unfaire Behandlung am Arbeitsplatz, häufiges Fehlen/erhöhte Fehlzeiten)
     Wenn ja: welche? ____

9. **Wie haben Sie reagiert?**

____

____

____

a. Abwehr/ Gegenwehr? ____

b. Was ist danach passiert? ____

c. Hat sich Ihre Situation danach verbessert oder verschlechtert? ❑ besser ❑ schlechter ❑ keine Änderung

d. Würden Sie erneut so handeln? ❑ ja ❑ nein

Sondern: ____

Falls keine Gegenwehr/Reaktion:

e. Können Sie mir einen Grund dafür nennen? ______________________________

f. Haben dabei Gefühle wie z.B. Angst, Scham, Hilflosigkeit eine Rolle gespielt?

____

g. Hat sich Ihre Situation danach verbessert oder verschlechtert? ❑ besser ❑ schlechter ❑ keine Änderung

h. Würden Sie erneut so handeln? ❑ ja ❑ nein

15. Als wie schwer haben Sie das Erlebte *damals* eingeschätzt? Skala 0 bis 10 ____

Als wie schwer schätzen Sie es *heute* ein? Skala 0 bis 10 ____

11. Sind Sie in Folge dieser Erfahrung in irgendeiner Art behandelt worden? ❑ ja ❑ nein

Falls ja: Wo? ❑ Krankenhaus ❑ Therapeut ❑ Hausarzt ❑ ________________________

10. Haben Sie jemandem von diesen Vorfällen/Ereignissen erzählt? ❑ ja ❑ nein

Falls ja: a) Wem haben Sie sich anvertraut?

❑ niemandem
❑ Familie
❑ Freunden
❑ ______________________
❑ Hausarzt/in , Krankenhausarzt
❑ sonstiges medizinisches Fachpersonal
❑ Beratungsstelle
❑ Opferhilfsorganisation
❑ Polizei

b) Wieso dieser Person/Institution? ______________________________

c) Wann haben Sie sich dieser Person/Organisation anvertraut? ______________________________

d) War das Reden hilfreich? ❑ ja ❑ nein

Können Sie das etwas genauer erklären?

____

____

12. Haben Sie wegen dieser Erfahrung jemals professionelle Hilfe aufgesucht? ❑ ja ❑ nein

Falls ja: a) Wo? ❑ Beratungsstelle ❑ Hilfsorganisation ❑ Kirche/Gemeinde ❑ Psychotherapeut ❑ Justiz (Polizei, Gericht, SA) ❑ ________________________

b) Wie lange waren Sie dort in „Beratung“? ______________________________

c) Woher kam der Kontakt/die Information? ______________________________

d) Wie lange hat es gedauert, bis Sie Hilfe gesucht haben? ______________________________

e) Hat Ihnen die Beratung dort geholfen? ❑ ja ❑ nein

Falls ja: Können Sie das etwas genauer erklären? ______________________________

Falls nein: Was hatten Sie sich anders vorgestellt/erhofft? ______________________________

Falls nein: Warum nicht? Was haben Sie befürchtet? ______________________________

13. Was hat Ihnen am ehesten geholfen, diese Erlebnisse zu verarbeiten?

❑ Freunde (Gespräche, Nähe)
❑ professionelle Hilfe
❑ Familie (Gespräche, Nähe)
❑ vergangene Zeit
❑ ____

14. Glauben Sie, dass es Gründe gibt, dass Ihnen dieses Ereignis zugestoßen ist? ❑ ja ❑ nein

Falls ja: Woran machen Sie das fest? ______________________________

____

D. Wirtschaftliche Gewalt

Sie haben vorhin angegeben, dass sie Opfer wirtschaftlicher Gewalt geworden sind. Können Sie dies etwas genauer beschreiben, bevor ich noch einige konkretere Fragen dazu stelle?

____

____

____

1. **Alter: Wie alt waren Sie?** ____________________
2. **Über welchen Zeitraum ist das passiert?** ____________________
3. **Wie häufig ist das vorgekommen?** ____________________
4. **Ort: Wo hat die Gewalterfahrung stattgefunden?**

   ❑ Arbeit
   ❑ zu Hause
   ❑ In der Stadt
   ❑ unterwegs
   ❑ in der Schule
   ❑ ____________________

5. **Täter: Wer hat die Gewalt ausgeübt?**

   ❑ Partner(in)/ ehemalige(r) Partner(in)
   ❑ Familie
   ❑ Bekannte
   ❑ ____________________
   ❑ Fremde
   ❑ Institutionen wie Polizei, Ordnungsamt, Arbeitsagentur
   ❑ berufliches Umfeld: Vorgesetzte, Kollegen

6. **Verhältnis: In welchem Verhältnis standen Sie zum Täter/zur Täterin?**

   ❑ Arbeitgeber
   ❑ Vorgesetzte
   ❑ Kollegen
   ❑ ____________________
   ❑ Familie
   ❑ Partner(in)/ ehemalige(r) Partner(in)
   ❑ Freunde/ Bekannte
   ❑ Fremde

7. **Abhängigkeit: Waren Sie auf irgendeine Art an diese Person gebunden?**

   ❑ emotional
   ❑ sozial
   ❑ wirtschaftlich
   ❑ ____________________

8. **Folgen: Gabe es …….. Probleme/Verletzungen aufgrund dieser Gewalterfahrung?**

   ❑ körperliche (Verletzungen, Krankheiten, dauerhafte/funktionelle Beeinträchtigungen)
   Wenn ja: welche? (Dokublatt Hessen beilegen!) ____________________
   ❑ psychische (Angst, Scham, Schuldgefühle, Rückzug, Wut)
   Wenn ja: welche? ____________________
   ❑ wirtschaftliche (Geldverlust, Verlust der Wohnung)
   Wenn ja: welche? ____________________
   ❑ soziale (sozialer Rückzug, Aufgabe von Freizeitaktivitäten, Spannungen im Familien- oder Freundeskreis)
   Wenn ja: welche? ____________________
   ❑ berufliche (Verlust der Arbeit, unfaire Behandlung am Arbeitsplatz, häufiges Fehlen/erhöhte Fehlzeiten)
   Wenn ja: welche? ____________________

9. **Wie haben Sie reagiert?**

____

____

____

a. Abwehr/ Gegenwehr? ____________________
b. Was ist danach passiert? ____________________
c. Hat sich Ihre Situation danach verbessert oder verschlechtert? ❑ besser ❑ schlechter ❑ keine Änderung
d. Würden Sie erneut so handeln? ❑ ja ❑ nein
Sondern: ____________________

Falls keine Gegenwehr/Reaktion:

e. Können Sie mir einen Grund dafür nennen? ____

f. Haben dabei Gefühle wie z.B. Angst, Scham, Hilflosigkeit eine Rolle gespielt?

____

g. Hat sich Ihre Situation danach verbessert oder verschlechtert? ❑ besser ❑ schlechter ❑ keine Änderung

h. Würden Sie erneut so handeln? ❑ ja ❑ nein

15. Als wie schwer haben Sie das Erlebte *damals* eingeschätzt? Skala 0 bis 10 ____
Als wie schwer schätzen Sie es *heute* ein? Skala 0 bis 10 ____

11. Sind Sie in Folge dieser Erfahrung in irgendeiner Art behandelt worden? ❑ ja ❑ nein

Falls ja: Wo? ❑ Krankenhaus ❑ Therapeut
❑ Hausarzt ❑ ________________________

10. Haben Sie jemandem von diesen Vorfällen/Ereignissen erzählt? ❑ ja ❑ nein

Falls ja: a) Wem haben Sie sich anvertraut?

❑ niemandem
❑ Familie
❑ Freunden
❑ ____________________
❑ Hausarzt/in , Krankenhausarzt
❑ sonstiges medizinisches Fachpersonal
❑ Beratungsstelle
❑ Opferhilfsorganisation
❑ Polizei

b) Wieso dieser Person/Institution? ____

c) Wann haben Sie sich dieser Person/Organisation anvertraut? ____________________________

d) War das Reden hilfreich? ❑ ja ❑ nein

Können Sie das etwas genauer erklären?

____

____

12. Haben Sie wegen dieser Erfahrung jemals professionelle Hilfe aufgesucht? ❑ ja ❑ nein

Falls ja: a) Wo? ❑ Beratungsstelle ❑ Hilfsorganisation ❑ Kirche/Gemeinde
❑ Psychotherapeut ❑ Justiz (Polizei, Gericht, SA) ❑ ________________________

b) Wie lange waren Sie dort in „Beratung“? ___________________________________

c) Woher kam der Kontakt/die Information? ____

d) Wie lange hat es gedauert, bis Sie Hilfe gesucht haben? _______________________________

e) Hat Ihnen die Beratung dort geholfen? ❑ ja ❑ nein

Falls ja: Können Sie das etwas genauer erklären? ______________________________________

Falls nein: Was hatten Sie sich anders vorgestellt/erhofft? _______________________________

Falls nein: Warum nicht? Was haben Sie befürchtet? ____

13. Was hat Ihnen am ehesten geholfen, diese Erlebnisse zu verarbeiten?

❑ Freunde (Gespräche, Nähe) ❑ professionelle Hilfe
❑ Familie (Gespräche, Nähe) ❑ vergangene Zeit
❑ ____

14. Glauben Sie, dass es Gründe gibt, dass Ihnen dieses Ereignis zugestoßen ist? ❑ ja ❑ nein

Falls ja: Woran machen Sie das fest? ____

____

MINI Einleitung

Im nächsten Teil führen wir ein sog. Klinisches Interview durch. Dabei geht es vor allem um die Frage, ob irgendwann in Ihrem Leben psychische Probleme bestanden haben und ggf. wie diese aussahen.

a) Gab es irgendwann mal eine Zeitspanne, in der Sie nicht fähig waren, zu arbeiten (>4 Wochen)?

❑ ja ❑ nein **Falls ja**: Wann war das? ______________________

Was war der Grund? ______________________

b) Waren Sie schon einmal in psychiatrischer/psychotherapeutischer Behandlung?

❑ ja ❑ nein **Falls ja:** Wann war das? ______________________

Was war der Grund? ______________________

c) Aus welchem Grund waren/sind Sie momentan in der Uniklinik in Behandlung?

____

- ❑ Patient
- ❑ Angehöriger
- ❑ Personal
- ❑ Student/in
- ❑ Blutspende
- ❑ Sonstiges

d) Nehmen Sie momentan Medikamente ein?

❑ ja ❑ nein **Falls ja:** Welche? ______________________

e) Wie viel Alkohol haben Sie ungefähr im letzten Monat getrunken?

____

f) Haben Sie im letzten Monat irgendwelche Drogen genommen?

❑ ja ❑ nein **Falls ja:** Welche? ______________________

g) Wie würden Sie Ihre Stimmung im letzten Monat einschätzen?
Skala 0 bis 10 ____

____

h) Gehen Sie derzeit einer Arbeit nach? (Mehrere Antworten möglich)

- ❑ Vollzeitstelle (mind. 30 Stunden/Woche)
- ❑ Teilzeitstelle (weniger als 30 Stunden/Woche)
- ❑ Ich arbeite ehrenamtlich (unbezahlt)
- ❑ Ich mache ein Praktikum (unbezahlt)
- ❑ Ich arbeite an einem geschützten Arbeitsplatz (z.B. Werkstatt)
- ❑ Ich arbeite zuhause (z.B. versorge Kinder oder Verwandte)
- ❑ Ich bin auf Arbeitssuche seit ______________
- ❑ ALG I
- ❑ ALG II
- ❑ Erwerbs- oder Berufsunfähigkeit seit ______________
- ❑ Student/in, Azubi
- ❑ Umschüler
- ❑ Renner/in, Pension, Vorruhestand
- ❑ andere ______________________________

Im Anschluss dann Screeningbogen und nötige Module…

Fragebögen:

- ❑ BDI-II
- ❑ BSI
- ❑ STAI
- ❑ STAXI
- ❑ SF-12 Zeitfenster 4 Wochen
- ❑ EQ-5D-5L
- ❑ WHO QOL bref
- ❑ F-SozU Version K-22
- ❑ SCI
- ❑ IES
- ❑ NEO-FFI-30
- ❑ ASQ
- ❑ ADP
- ❑ MINI
